# Utilization of phosphate-responsive promoters for auto-inducible management of biotechnological processes based on cyanobacteria

**DOI:** 10.1016/j.mec.2026.e00278

**Published:** 2026-05-22

**Authors:** Marvin Amadeus Itzenhäuser, Franziska Hufnagel, Carla Klemm, Kevin Otec, Minmin Pan, Stephan Klähn

**Affiliations:** a Department of Solar Materials Biotechnology, Helmholtz Centre for Environmental Research – UFZ, Permoserstr. 15, 04318, Leipzig, Germany; b Department of Microbial Biotechnology, Helmholtz Centre for Environmental Research – UFZ, Permoserstr. 15, 04318, Leipzig, Germany

**Keywords:** Synthetic biology, Promoter, Gene expression, Phosphate signaling, Cyanobacteria, (photo)biotechnology

## Abstract

Controllable gene expression is essential in microbial biotechnology, yet most systems rely on costly external inducers that limit large-scale applicability. Here, we employed phosphate-responsive promoters controlled by the SphS–SphR phosphate-sensing two-component system as auto-inducible expression systems in cyanobacteria. Specifically, we characterized the promoters of *phoA*, *sphX*, and *pstS2,* which are activated under low phosphate availability and achieved induction folds of up to 13, as well as P*urtA* and a synthetic promoter P*P*_*i*_*-neg*, which are repressed under these conditions. Replacement of the ribosome binding site in the native promoter systems further expanded the accessible range of expression levels, with context-dependent increases or decreases depending on the promoter. By systematically adjusting the phosphate concentration and cell density, the timing of gene expression could be precisely controlled. Notably, intracellular phosphate storage during early growth enables transient buffering of external depletion, allowing promoter activation prior to growth limitation. This, in turn, enables their use in continuously growing cultures. As a proof of concept, we established a sucrose production process that autonomously transitioned from a growth phase with basal production to a production phase with a 5.5-fold higher sucrose titre, triggered by phosphate depletion during cultivation. Overall, this auto-inducible expression system expands the cyanobacterial genetic toolbox and enhances the applicability of cyanobacteria in scalable production processes.

## Highlights

• Phosphate-responsive promoters were characterized in cyanobacteria.
• A synthetic promoter repressed under low phosphate was designed.
• Induction of the promoters can be modulated via the phosphate concentration.
• As a proof of concept, an auto-inducible sucrose production process was established.
• Conservation of SphR allows transfer to other cyanobacteria.

## 1 Introduction

Bacterial growth is strongly influenced by nutrient availability, which can fluctuate rapidly due to environmental changes and cellular uptake. To cope with this variability, bacteria employ diverse regulatory systems that coordinate the expression of genes involved in nutrient acquisition and assimilation (Dworkin and Harwood, 2022; Padilla-Vaca et al., 2017). Phosphorus, typically assimilated as inorganic phosphate (P_i_), is an essential component of nucleotides, nucleic acid, and phospholipids. In freshwater environments, P_i_ often limits the growth of cyanobacteria, that perform oxygenic photosynthesis and hence are major primary producers in those habitats. To adapt to fluctuating P_i_ availability, many cyanobacterial species exhibit luxury uptake – transporting P_i_ at rates exceeding immediate growth requirements – and store it as polyphosphate granules or nucleic acids (Griese et al., 2011). This strategy helps sustaining growth during P_i_-depleted periods (Xiao et al., 2022).

A common mechanism for environmental sensing in bacteria involves two-component regulatory systems, which consist of a sensor kinase and cognate response regulator. These systems allow cells to perceive extracellular signals and modulate gene expression accordingly (Groisman, 2016; Mitrophanov and Groisman, 2008; Padilla-Vaca et al., 2017). In the model organism *Escherichia coli* (hereinafter *E. coli*), phosphate signaling is mediated by the PhoR–PhoB two-component system that coordinates the expression of genes under phosphate-limiting conditions, e.g. the genes for the phosphate transporter system PstABC (Santos-Beneit, 2015). Homologous systems are also found in many other bacteria including cyanobacteria, where it is designated as SphS–SphR (Hirani et al., 2001; Santos-Beneit, 2015; Suzuki et al., 2004). The SphS-SphR system has been studied in detail in the model cyanobacterium *Synechocystis* sp. PCC 6803 (hereinafter *Synechocystis*) where the phosphorylation status of the histidine kinase SphS is modulated in accordance with external P_i_ concentrations. Under P_i_-limiting conditions SphS undergoes autophosphorylation, at high P_i_ SphS remains unphosphorylated. Although the precise molecular mechanism of P_i_ sensing is not fully elucidated, it likely involves interactions between the N-terminal, membrane-associated region of SphS and the Pst1 phosphate transporter complex, as well as negative regulation by SphU (Burut-Archanai et al., 2009; Juntarajumnong et al., 2007; Kimura et al., 2009). Subsequent to auto-phosphorylation, SphS transfers its phosphate group to its response regulator SphR. Phosphorylated SphR is then able to induce transcription from promoters containing SphR-binding motifs (Suzuki et al., 2004). Via this regulatory circuit, *Synechocystis* upregulates genes involved in phosphate acquisition and mobilization in response to P_i_ limitation. These include genes encoding high-affinity phosphate-binding proteins (e.g., SphX, PstS), P_i_ transport systems (PstABC), and enzymes that liberate phosphate from organic sources, such as the alkaline phosphatase PhoA and the extracellular nuclease NucH (Solovchenko et al., 2020; Sun et al., 2024; Suzuki et al., 2004). Homologous SphS-SphR systems are conserved across most cyanobacteria, including *Synechococcus elongatus* PCC 7942 (hereinafter *Synechococcus elongatus*), suggesting a similar regulatory mechanism in other strains (Nagaya et al., 1994; Su et al., 2007).

Beyond their ecological importance, cyanobacteria have attracted growing interest as platforms for sustainable biotechnological production. Their photoautotrophic lifestyle, combined with the genetic accessibility of strains such as *Synechocystis*, enables light-driven, CO_2_-based production of chemicals, fuels, or hydrogen - processes that are considered sustainable and CO_2_-neutral (Appel et al., 2020; Santos-Merino et al., 2023; Sarsekeyeva et al., 2015; Till et al., 2020). Achieving high productivity in such systems requires precise control over cellular metabolism to direct resources toward product formation (Toepel et al., 2023). Consequently, the development of gene expression systems that enable control over metabolically engineered enzymatic activities or entire pathways is a critical prerequisite for advancing cyanobacterial biotechnology.

While a broad repertoire of inducible promoters is available in well-established chemoheterotrophic hosts such as *E. coli*, *Bacillus subtilis*, or *Corynebacterium glutamicum* (Balzer et al., 2013; Ejaz et al., 2022; Lee and Kim, 2018)*,* these systems are typically not directly transferable to cyanobacteria due to fundamental differences in transcriptional regulation including sigma factor usage (Imamura and Asayama, 2009). Consequently, the molecular toolbox for controlled gene expression in cyanobacteria remains more limited. In recent years, several inducible promoter systems have been adapted for cyanobacterial strains for example by adjusting the position of the operators in case of the Isopropyl β-D-1-thiogalactopyranoside (IPTG) inducible promoter (Camsund et al., 2014) or strongly expressing a heterologous transcription factor to facilitate rhamnose inducible gene expression (Kelly et al., 2018). Other systems for inducible expression were specifically developed for cyanobacteria based on intrinsic regulatory systems such as heavy metal-responsive promoters (Peca et al., 2007; Perez et al., 2017), or an intrinsic guanidine-riboswitch (Itzenhäuser et al., 2025). However, most inducers introduce high additional costs and process complexity - factors that become increasingly significant in large-scale or continuous operations. Even in processes with chemoheterotrophs, where rich carbon sources are used, inducers can represent the most expensive medium component despite their low concentrations (Cardoso et al., 2020). By employing the guanidine-responsive riboswitch, gene expression can be triggered by changing the nitrogen source in cyanobacterial applications, which comes with comparably low costs (Itzenhäuser et al., 2025).

To reduce these limitations further, auto-inducible promoter systems, which are activated by internal or culture-related signals rather than external inducer addition, are attractive for industrial applications. In classical hosts, such systems have been developed based on diverse regulatory mechanisms, including stress-responsive (Kim et al., 2016; Panahi et al., 2014), or stationary phase specific promoters (Anilionyte et al., 2018; Jaishankar and Srivastava, 2020; Miksch et al., 2005; Retnoningrum et al., 2019), as well as quorum-sensing-based regulation (Cheng et al., 2016; Cui et al., 2024; Lee et al., 2010; Meyers et al., 2019; Nocadello and Swennen, 2012; Sun et al., 2020). In addition, promoters activated by nutrient limitation, particularly those of the Pho regulon responding to phosphate depletion, have been successfully employed for auto-inducible expression (Gundinger et al., 2022; Kerovuo et al., 2000; Nguyen et al., 2019). In contrast, only two strategies to achieve auto-induction have been reported in cyanobacteria. One employed a growth phase-dependent promoter in *Synechococcus* sp. PCC 7002, while another introduced a heterologous quorum-sensing system into *Synechococcus elongatus* to achieve cell-density-dependent induction (Kokarakis et al., 2025; Madsen et al., 2021).

**Fig. 1:**
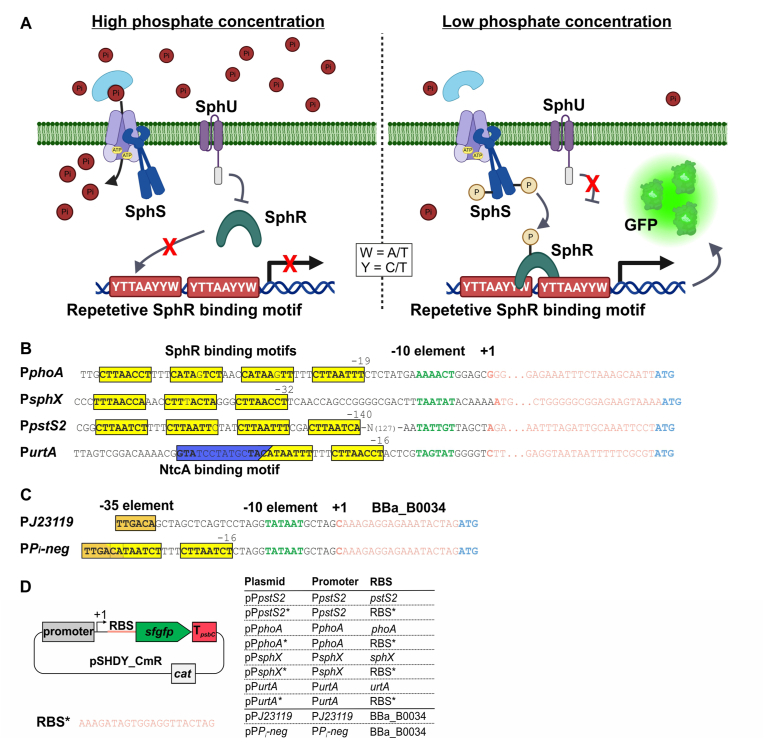
Design of reporter strains for phosphate-dependent auto-inducible promoters based on the SphS-SphR signalin**g cascade.** (A) Diagram illustrating the mechanism of phosphate-dependent reporter gene expression based on the SphS-SphR system. (B) Sequence analysis of four native promoters and RBS regions of the Pho-regulon. Sequences that resemble the SphR-motif were marked with yellow boxes with the nucleotides directly resembling the SphR-motif shown in bold. A NtcA binding motif is highlighted by a blue box. The distance of the first nucleotide of an identified Pho-box from the TSS (+1) is given above the respective SphR-motif. TSS were derived from Kopf et al. (2014). (C) Design of a synthetic promoter sequence based on a P*J23119* scaffold to enable repression of gene expression under P limitation (P*P*_*i*_*-neg*). (D) Design of replicative plasmids to drive phosphate-controlled reporter gene expression in *Synechocystis*. The promoter sequences shown in (B) and (C) were introduced into a reporter plasmid based on pSHDY where they were fused to the coding sequence of *sfgfp* either with their native RBS or RBS∗. Panel A has been created in BioRender. Klähn, S. (2026) https://BioRender.com/8qonhrl.

In this study, we explored an alternative strategy by coupling gene expression to the native phosphate-sensing two-component system SphS–SphR to enable auto-induction in *Synechocystis* (see Fig. 1). We characterized the phosphate-responsive expression of four native promoters from the Pho regulon and designed a synthetic promoter containing Pho boxes. By combining these promoter elements with either native or synthetic ribosome binding sites (RBS), we constructed a promoter library with varying expression strengths, induction timing, and regulatory profiles. Because promoter activation is directly linked to P_i_ availability, auto-induction can be precisely controlled by adjusting the P_i_ concentration in the medium. Notably, due to the natural luxury uptake of P_i_ by *Synechocystis,* external phosphate depletion can be transiently buffered, potentially allowing induction to occur before significant growth limitation sets in. As a proof of concept, we applied one of the characterized promoters to drive sucrose biosynthesis and demonstrated a production process that autonomously transitions from a growth to a production phase. Finally, we show that the conservation of the SphS–SphR system across cyanobacterial species enables the direct transfer of this auto-inducible expression system to other strains.

## 2 Material and methods

### 2.1 Strains and culture conditions

*Synechocystis* and *Synechococcus elongatus* were obtained from the Pasteur Culture Collection. By default, cyanobacterial strains were grown in modified BG11 liquid medium or on plates of BG11 medium solidified with 1.5 % agarose as described before (Itzenhäuser et al., 2025). To adjust the phosphate concentration in the medium, either the amount of dipotassium phosphate was set to the stated value or, if exceeding 0.5 mM, replaced by a dipotassium phosphate – monosodium phosphate buffer set to pH 8.2. For sucrose production, the medium was either prepared with additional 200 mM NaCl or supplemented with 500 mM NaCl to induce a salt shock. If grown in shaking flasks, cultures were incubated in a Multitron Pro (INFORS HT) set to 30 °C, 50 μE∗m^−2^∗s^−1^, ambient CO_2_, 70 % humidity, and 150 rpm. The biomass density was analyzed by external measurements of the optical density at 750 nm (OD_750_) using the Genesys 150 UV/Vis spectrophotometer (Thermo scientific). For continuous monitoring of GFP fluorescence, cultures were grown in the Biolector XT microbioreactor equipped with the light array module (Beckmann Coulter) in 48-well flower plates with a culture volume of 1 mL, under conditions of gassing with 50 mL/min CO_2_ enriched (1 %) air, a combination of red and blue lighting of overall 200 μE∗m^−2^∗s^−1^, 30 °C, and 1200 rpm shaking. In the Biolector, biomass density was monitored via scattered light measurements at 730 nm (SL_730_). Continuous cultures were implemented in Multicultivator MC1000 bubble column reactors (Photon Systems Instruments) with on-line culture density measurement at 730 nm (OD_730_) equipped with the turbidostat system. In this case conditions were set to 30 °C, 50 μE∗m^−2^∗s^−1^, and gassing with 1 % CO_2_ enriched air.

For cloning, *E*. *coli* TOP10 were grown in lysogeny broth (LB) medium or on respective agar-solidified plates. To select and sustain recombinant strains, the media were supplemented with 100 μg/mL ampicillin (Amp), 50 μg/mL kanamycin (Kan), or 15 μg/mL chloramphenicol (Cm).

### 2.2 Strain construction

Genetic constructs were created using polymerase chain reaction (PCR) with Q5® polymerase (New England Biolabs) using the primers given in Table S1 and assembled into plasmids using a previously described modular Golden-Gate cloning (GGC) system (Itzenhäuser et al., 2025; Lupacchini et al., 2025). The employed plasmids and their construction are described in Table S2. Electro-competent *E. coli* TOP10 were transformed as routine (Green and Sambrook, 2020). Plasmids were confirmed using Sanger sequencing. Chromosomal deletions or integrations in *Synechocystis* or *Synechococcus elongatus* were achieved by homologous recombination and subsequent selection on respective antibiotics (Clerico et al., 2007; Kufryk et al., 2002). Replicative plasmids were introduced into *Synechocystis* by electroporation as described before (Brandenburg et al., 2021; Opel et al., 2022). Successful transformation and segregation were verified by PCR using GoTaq® PCR master mix (Promega).

### 2.3 Analysis of GFP fluorescence

GFP fluorescence was either measured externally in an Infinite 200 PRO microplate reader (Tecan) or on-line in the Biolector XT. For external GFP analysis, cultures were diluted to an OD_750_ of 0.25 in 200 μL and transferred into opaque black flat microtiter 96-well plates (Nunc) in triplicates. The optical measurement was performed as described previously and normalized to the OD_750_ (Opel et al., 2022). In case of the automated GFP measurement in the Biolector XT, the machine was equipped with the GFP/Gemini filter and the respective fluorescence signal of each well was recorded with a gain of 5 at short intervals. A background correction was performed by subtracting the average signal of wildtype cultures at each timepoint.

### 2.4 Measurement of phosphate concentration

Remaining phosphate in the medium was quantified from the 1:10 diluted supernatant of a 2 ml sample of the culture by ion chromatography on the ICS-6000 (ThermoFisher) equipped with a Dionex IonPac AS11-HC-4μm 2 × 250 mm column. An EGC 500 KOH cartridge (eluent) was employed to generate the isocratic eluent flow of 0.38 mL/min with a concentration of 18 mM for 8 min, followed by a 1.5 min phase at 40 mM, and 0.5 min at 1 mM.

### 2.5 Sucrose extraction and HPLC analysis

The intracellular sucrose concentration was measured from the cell pellet of a 2 ml sample of the cell culture. Therefore, the pellet was re-suspended in 150 μl ice-cooled 80 % ethanol and homogenized using glass beads in the Precellys Evolution Touch (3 × 20 s; 10,000 rpm). Subsequently, the homogenized sample was incubated at 55 °C for 10 min and then centrifuged for 45 min at 16,000 xG. The resulting pellet was dried using a vacuum concentrator and re-suspended in 200 μL water. After treatment for 5 min in an ultrasonic bath and centrifugation for 10 min at 16,000 xG, the supernatant was subjected to a HPLC analysis on the Vanquish Flex (ThermoFisher) equipped with an Agilent Hi-Plex Ligand Exchange (Hi-Plex H, 300 × 7.7 mm, 8 μM) column kept at 15 °C. Water was used as the mobile phase at a flow rate of 0.6 mL/min. A refractive index detector (Refractomax521) at 35 °C was used for detection.

## 3 Results

### 3.1 Design of synthetic promoters responsive to phosphate

In *Synechocystis*, several genes have been identified to be controlled by P_i_ availability via the two-component system SphS-SphR, which are nevertheless designated as Pho regulon. The corresponding promoters are targeted by the phosphorylated transcription factor SphR, which binds to specific DNA motifs (SphR binding motifs). These binding sites are characterized by repeats of the consensus motif YTTAAYYW (Y = C or T; W = A or T) (Suzuki et al., 2004). Most genes of the Pho regulon are positively regulated under P_i_ limitation, as they encode proteins involved in phosphate acquisition and transport. The only known exception is *urtA*, which is repressed under P_i_ limitation by SphR binding (Suzuki et al., 2004). To employ the regulation of the SphS-SphR system, the promoters of the genes from the Pho regulon were analyzed by fusing them to a reporter gene and following its transcription in different phosphate concentrations (Fig. 1A). Detailed analyses of the promoter sequences revealed the presence of two to four SphR motifs, typically separated by a short three-nucleotide spacer, located upstream of the transcriptional start site (TSS). In positively regulated promoters such as P*phoA*, P*sphX*, or P*pstS2*, these Pho boxes either overlap the −35 region or are positioned further upstream (Fig. 1B). In contrast, in the negatively regulated promoter P*urtA*, two Pho boxes are located close to the −10 region, potentially interfering with σ-factor binding and thereby repressing transcription when SphR is bound (Fig. 1B).

Native promoters could in principle be used directly for P_i_-dependent gene regulation by fusing them to a target gene. However, transcription from native promoters can be affected by additional regulatory mechanisms. For example, *PurtA* is also regulated by the nitrogen starvation-responsive transcription factor NtcA (Giner-Lamia et al., 2017). To avoid such cross-regulation, a synthetic SphR-dependent promoter was designed based on the known arrangement of SphR motifs, which potentially allows for controlled negative regulation while minimizing interference with other regulatory networks. As a scaffold, the constitutive σ^70^-dependent core promoter P*J23119* was chosen, as it has been shown to drive strong and constitutive gene expression in *Synechocystis* and does not contain additional transcription factor binding sites (Vasudevan et al., 2019). For the design of a potentially negatively regulated promoter (P*P*_*i*_*-neg*), two SphR motifs repeats were introduced between the −35 and −10 elements, partially overlapping the −35 region. This configuration, inspired by *PurtA*, was expected to allow repressive regulation by SphR through competition with σ^70^ binding (Fig. 1C).

As both transcription initiation and translation efficiency mainly determine overall performance of gene expression, the ribosome binding site (RBS) was also considered previously (Thiel et al., 2018). While the P*J23119*-based synthetic constructs use the standardized synthetic RBS BBa_B0034 (http://parts.igem.org), native SphR-dependent genes are associated with their corresponding RBS sequences present upstream of the open reading frame. To evaluate whether translation can be optimized without affecting transcriptional regulation, the native RBSs were optionally replaced with RBS∗, a synthetic sequence optimized for *Synechocystis* (Englund et al., 2016). These additionally designed variants were designated as P*phoA*∗, P*sphX*∗, P*pstS2*∗ and P*urtA*∗, respectively.

**Fig. 2:**
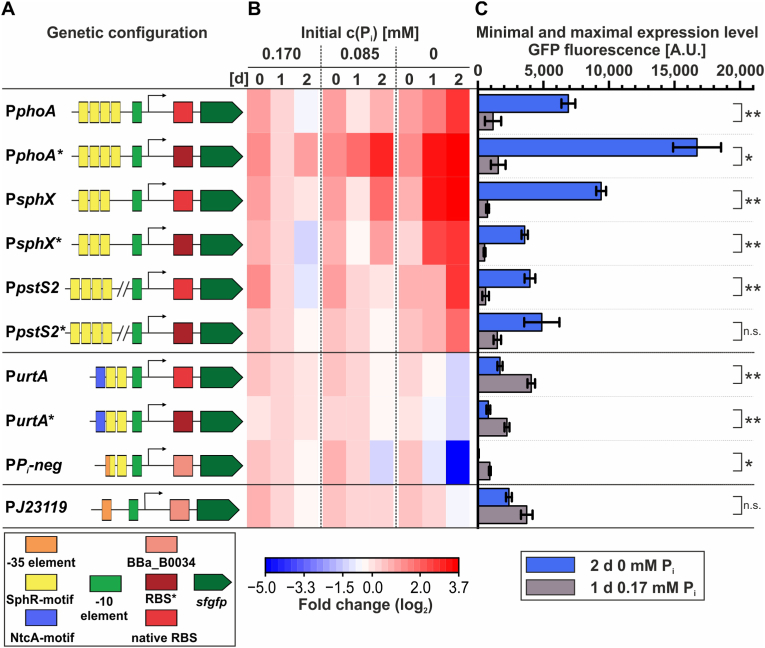
Characterization of phosphate-responsive promoters*.* Expression of a reporter gene was analyzed in *Synechocystis* strains carrying plasmids with the respective promoters fused to a *sfgfp* coding sequence. Cultures were transferred from standard BG11 medium containing 0.17 mM phosphate to media with either 0.17 mM, 0.085 mM, or 0 mM phosphate and incubated for two days. Measurements represent three biological replicates from independent colonies with three technical replicates for each data point. Fluorescence values of a strain harboring an empty vector were subtracted as background. (A) Schematic representation of the organization of the analyzed expression systems. (B) Fold change of the GFP fluorescence level over the two days in the different phosphate concentrations relative to the level after one day in standard BG11 medium. Complete disappearance of a GFP signal was set to -5-fold. (C) Absolute GFP fluorescence after one day in standard BG11 medium (the reference point for the calculation of the fold changes) and after two days in phosphate-free medium (the point with the strongest activation or repression). Data are the mean±standard error based on three biological replicates. Statistical significance between conditions (induced vs. non-induced) was assessed using Welch's *t*-test. Significance levels are indicated as follows: p > 0.05 (n.s.), p < 0.05 (∗), p < 0.01 (∗∗), p < 0.001 (∗∗∗). Absolute fluorescence values for each timepoint in each medium are displayed in Fig. S1.

To evaluate all designed promoters for their potential as auto-inducible expression systems in cyanobacteria, each promoter variant was fused to the coding sequence of the Superfolder GFP (*sfgfp*) on a pSHDY backbone, which enables extrachromosomal replication in *Synechocystis* (Behle and Axmann, 2022). For comparison reasons, also native SphR-dependent promoters were considered. P*P*_*i*_*-neg* was fused to *sfgfp* together with BBa_B0034, while the native promoters of *phoA*, *sphX*, *pstS2*, and *urtA* were fused either with their native 5′ UTR or with the native RBS replaced by RBS∗ (Fig. 1D). A schematic overview of each designed genetic configuration is given in Fig. 2.

### 3.2 Functional characterization of native and synthetic phosphate-responsive promoters

During cultivation, the phosphate concentration in the culture medium decreases due to cellular uptake. It has previously been indicated that phosphate is the major limiting factor when growing *Synechocystis* in BG11 medium (David et al., 2018). This natural phosphate depletion can therefore be used to achieve auto-induction of gene expression when coupled to phosphate availability. To test whether the designed expression systems respond to different phosphate concentrations, GFP fluorescence was measured after transferring cells from standard BG11 medium (0.17 mM phosphate) into either (i) standard BG11 (control), (ii) BG11 containing half the phosphate concentration (0.085 mM), or (iii) phosphate-free BG11, representing progressive stages of phosphate limitation. Under these conditions, all strains harbouring fusions of *sfgfp* with the promoters P*phoA*, P*sphX*, P*pstS2* showed increased GFP fluorescence, when cultivated in phosphate-free medium for one or more days (Fig. 2), which is in agreement with a positive regulation by SphR. In contrast, the strain with the P*urtA*::*sfgfp* fusion showed decreased GFP fluorescence, consistent with repression by SphR-binding (Fig. 2). This was also observed for the synthetic promoter P*P*_*i*_*-neg*, but with an even more drastic repression, reducing the signal of the reporter gene below detection limits after two days in phosphate-free medium. Consistent with the absence of SphR binding motifs, the constitutive J23119 promoter led to no phosphate-dependent expression pattern of the *sfgfp* gene fused to it (Fig. 2). The absolute expression levels obtained with the different constructs varied considerably (Fig. 2C). Replacement of the native RBS with the synthetic RBS∗ increased *sfgfp* expression mediated by P*phoA* more than twofold, while for the corresponding construct design P*pstS2∗* only a slight enhancement was achieved, and P*sphX∗* and P*urtA∗* displayed reduced gene expression (Fig. 2).

The promoters also differed in their response dynamics to intermediate phosphate concentrations. For example, P*phoA∗* reached 73 % of its maximum activity (obtained in phosphate-free BG11) already in medium containing half the phosphate concentration after two days (Fig. 2C, Fig. S1). In contrast, P*pstS2* showed no activity increase above baseline under the same conditions within two days even though it is clearly induced in phosphate-free medium (Fig. 2C, Fig. S1). Based on these response profiles, promoters were categorized into three functional groups: (i) P*P*_*i*_*-neg*, P*urtA*∗, P*urtA*, and P*phoA∗* can be considered as early promoters, with ≥25 % of maximal expression or repression already achieved under half-phosphate conditions in two days, (ii) P*sphX*, P*sphX*∗, and P*phoA* as midway promoters, with 5–24 % of maximal activity, and (iii) P*pstS2* and P*pstS2∗* as late promoters, with <5 % of maximal expression under these conditions.

A concentration-dependent response to phosphate indicates that expression is regulated by the SphS–SphR system as intended. To verify this, the synthetic promoter P*P*_*i*_*-neg* and the two strongest inducible promoters, P*sphX* and P*phoA∗*, were also tested in a Δ*sphS* mutant. Without the regulation of SphS, the *sfgfp* expression from both positively controlled promoters was drastically reduced, and sensitivity to phosphate concentration was lost (Fig. S2). In case of the negatively controlled P*P*_*i*_*-neg* the initial expression levels were comparable between WT and Δ*sphS* mutant, but the phosphate-limitation induced repression was eliminated (Fig. S2). This underlines direct dependence of *sfgfp* expression on the SphS–SphR system.

Taken together, the tested promoter constructs constitute a diverse set of phosphate-responsive expression systems with distinct expression strengths, induction ratios, and activation thresholds (Fig. 2). In particular, P*sphX* and P*phoA∗* represent promising promoters for applications where strong gene expression is desired only in later cultivation phases, as both achieved induction ratios exceeding tenfold compared with phosphate-replete conditions.

### 3.3 Induction timepoints can be shifted by modulating initial phosphate concentrations

In biotechnological processes using whole-cell catalysts, the optimal timepoint for target gene expression can vary depending on the process and its stage. Promoters with different response characteristics, such as early or late induction, can therefore be useful to achieve the desired timing of expression. Because the activity of the presented promoters is directly coupled to phosphate concentration, it should also be possible to control induction of gene expression by adjusting the phosphate level in the growth medium.

**Fig. 3:**
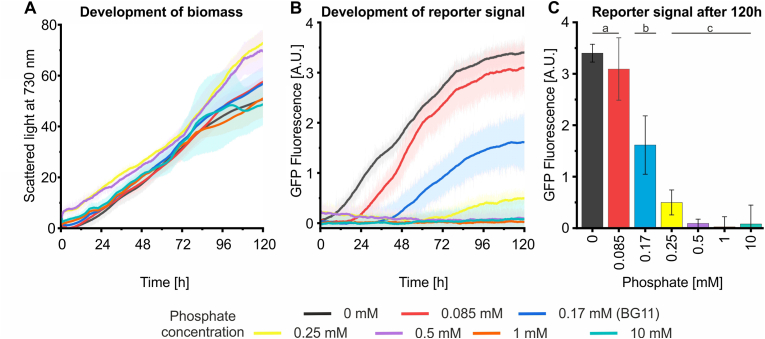
Control of induction timing via phosphate conc**entration.***Synechocystis* carrying a plasmid with the P*phoA*∗*sfgfp* construct was cultivated in BG11 media containing different phosphate concentrations (0 – 10 mM) in a Biolector XT® microbioreactor equipped with the light-array-module for illumination. Cultures were grown in 1 ml volumes in a 48-well plate with 4-14 replicates per condition. Fluorescence signals from a parallel wild-type culture were subtracted as background. Shaded areas and error bars indicate standard deviations based on four to eight replicates. (A) Culture density, (B) GFP fluorescence were monitored by automated optical measurements in short intervals. (C) The GFP fluorescence signal after 120 h of cultivation was extracted from the data in (B). Differences between groups were analyzed using one-way ANOVA followed by Tukey's post hoc test. Groups not sharing a common letter are significantly different (p < 0.05).

To demonstrate this process management potential, the *Synechocystis* strain expressing *sfgfp* under the control of P*phoA∗* was cultivated in BG11 media containing phosphate concentrations ranging from 0 to 10 mM. Culture density and GFP fluorescence were monitored continuously. During the first three days, no difference in growth was observed among the cultures, even in phosphate-free medium, indicating that *Synechocystis* can perform several cell divisions using intracellular phosphate reserves (Fig. 3A). At later stages, growth slowed down in both phosphate-free and high-phosphate (1 and 10 mM) media compared with standard BG11 (0.17 mM). In contrast, cultures with slightly higher phosphate concentrations (0.25 and 0.5 mM) grew faster after approximately 3.5 days.

Consistent with previous results, immediate and continuous evolution of GFP fluorescence was observed in phosphate-free medium, which is a result of *sfgfp* expression driven by the P*phoA∗* promoter configuration (Fig. 3B). By adjusting the initial phosphate concentration, the onset of induction could be systematically delayed: addition of 0.085 mM phosphate (half the standard concentration) postponed induction of P*phoA∗* by roughly 20 h, while cultures in standard BG11 (0.17 mM) initiated expression after about 36 h. Increasing the phosphate concentration to 0.25 mM delayed induction to 72 h, and no induction was observed within 120 h when phosphate concentrations of 0.5 mM or higher were used (Fig. 3B). Notably, the maximum expression level achieved was inversely correlated with the initial phosphate concentration, as fluorescence in phosphate-containing media did not reach the same intensity as under phosphate-depleted conditions during the 120 h of this cultivation (Fig. 3C).

### 3.4 Tuning promoter activation through cell density in continuous cultivation

The phosphate concentration in the culture medium changes during cultivation in correlation with the cell density (David et al., 2018). Therefore, it takes different times until the concentration falls below the threshold for promoter activation when the cultivation is started in presence of different phosphate concentrations (Fig. 3). In a continuous cultivation the culture is constantly diluted to keep a constant cell density. Thereby, the volumetric rate of dilution with fresh medium is higher when a lower cell density is targeted. In consequence, less of the nutrients from medium such as phosphate is used for cell growth until it is removed from the cultivation again and a steady state concentration of these nutrients arises which is inversely correlated to the cell density. This could be used with the presented phosphate-responsive promoters, additionally to the initial phosphate concentration of the medium, to manage induction of target genes in continuous cultivations by setting the targeted density in a way that the steady-state phosphate concentration falls below the threshold for promoter activation.

**Fig. 4:**
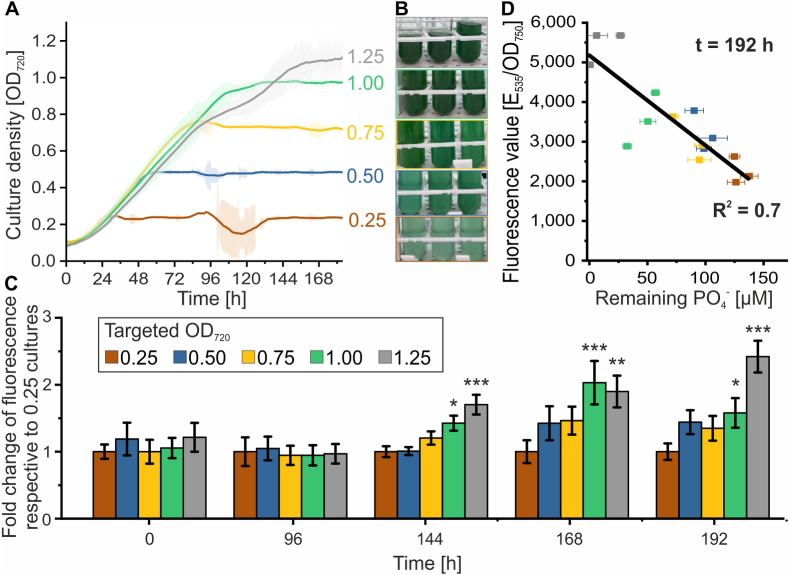
Control of gene expression induction via cell density in continuous **cultures.** (A) Growth of *Synechocystis* carrying a plasmid with the P*phoA*∗::*sfgfp* construct was cultivated in BG11 medium in a Multicultivator MC-1000 operated in turbidostatic mode using peristaltic pumps for automatic dilution. Culture density was monitored automatically; because measurements were taken without concurrent dilution, readings at higher optical densities are not linear. Addition of fresh BG11 medium was automatically triggered when the culture exceeded the set optical density. Different target cell densities were maintained in separate vials, each in triplicates. Data are the mean ± SD of triplicate cultures. (B) Photographs of the vials after nine days of cultivation in turbidostatic mode. (C) Ratios of OD-normalized GFP fluorescence in each condition relative to cultures maintained at OD_720_ = 0.25. Error bars represent propagated standard errors calculated from the three biological replicates per condition and the reference cultures. For each time point, differences between conditions were analyzed using one-way ANOVA followed by Dunnett's post hoc test, comparing each condition to the reference condition (OD_720_ = 0.25). Significant differences are indicated as p > 0.05 (n.s.), p < 0.05 (∗), p < 0.01 (∗∗), p < 0.001 (∗∗∗). (D) Correlation between the residual phosphate concentration in the medium and GFP fluorescence measured after eight days of continuous cultivation. Phosphate concentrations were determined by ion chromatography from culture supernatants after centrifugation. Error bars represent standard deviations from three independent samples for phosphate determination or from four technical replicates for fluorescence measurement. Fluorescence values represent uncorrected signals in the GFP channel, and all derived quantities (e.g., normalized ratios) were calculated from these raw data.

This management strategy was again tested with the P*phoA*∗-controlled *sfgfp* expression in *Synechocystis*. Therefore, cultures were grown to and kept at different targeted optical densities by automatic dilution in BG11 medium. Thereby, the different cell densities ranging from OD_720_ 0.25 to 1.25 were reached gradually and then kept for over a week (Fig. 4A and B). Moreover, the GFP fluorescence was measured regularly and did not differ between the different cultures within the first four days, when ODs were ≤0.75. However, an increase in GFP fluorescence was observed in the cultures exceeding this OD in the following measurements. Especially the cultures grown to the highest OD of 1.25 showed a substantial induction after 6-7 days (Fig. 4C). The maximum GFP fluorescence for each culture was obtained after 8 days and clearly correlated with the remaining phosphate concentration in the medium (Fig. 4C). These results demonstrate that the timing and extent of induction from phosphate-responsive promoters can be precisely managed in continuous cultures by adjusting the targeted cell density.

### 3.5 Auto-induction by PphoA∗ enables two stage production process for sucrose

In many biotechnological processes allocation of cellular resources is optimized by differentiating a growth from a production stage (Burg et al., 2016). In the growth phase, gene expression for target enzymes is not activated and resources can be optimally used to grow the biomass used as a catalyst later. The subsequent production stage profits from the established concentration of the catalyst and hence resources can be allocated to product formation by induction of the expression of respective enzymes. The potential of the presented phosphate-responsive promoters to facilitate automatic transition between such stages via their tunable auto-induction capacity was further demonstrated using P*phoA*∗-controlled sucrose production. To achieve such a process, the *spsA* gene was overexpressed in *Synechocystis* from a plasmid under the regulation of P*phoA*∗ (pSHDY_P*phoA*∗::*spsA*). The sucrose-phosphate synthase (SPS) encoded by *spsA* catalyzes the rate-limiting step of sucrose production in cyanobacteria (Du et al., 2013). Because normally sucrose is only transiently produced in *Synechocystis* as a response to salt-stress and is decomposed and replaced by other compatible solutes later, a mutant lacking the invertase, which is responsible for sucrose degradation, was used in the process (*Synechocystis* Δ*inv*) (Fig. S3) (Kirsch et al., 2018).

**Fig. 5:**
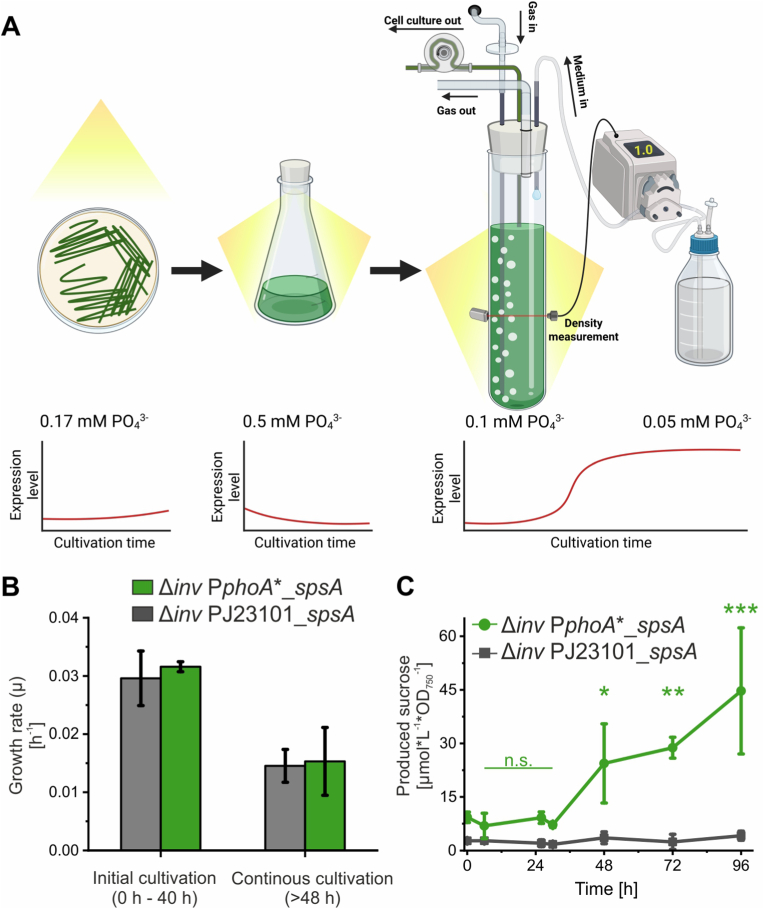
Automatic phase-transition in a two-stage production process for the osmolyt**e sucrose.** (A) Schematic representation of the process from precultures with high phosphate to the two-stage process with an initial growth phase and a continuous cultivation phase with systematically reduced phosphate concentrations. By managing the phosphate concentration in the media used at different stages of a production process and the density in the final continuous cultivation, the P*phoA*∗ expression system could be controlled to auto-induce sucrose production. Anticipated promoter activity in the different process steps is given based on the previous experiments. Panel A has been created in BioRender. Klähn, S. (2026) https://BioRender.com/e14fmvw (B-C) *Synechocystis* strains with deleted invertase gene carrying plasmids for the overexpression of the sucrose phosphate synthase gene (*spsA*) either controlled by the constitutive J23101 promoter or the *phoA*∗ promoter were cultivated according to the process design. (B) Growth rates of the two strains in the initial growth phase and the continuous cultivation in the Multicultivator bubble-column reactors. Sequential dilution to achieve continuous cultivation was triggered at ∼48 h when the cultures reached the set density of 1.0 No significant differences in growth rates were detected between strains (Welch's *t*-test). However, equivalence within predefined bounds of ±10% could not be demonstrated either (TOST). (C) Sucrose concentration of the two strains over the time course of the production process. Data represent the average and standard deviation of three biological replicates. Data were analyzed using a linear mixed-effects model with strain and time as fixed effects and replicate as a random effect. Pairwise comparisons relative to t0 were performed with Holm adjustment. Significance levels are indicated as p > 0.05 (n.s.), p < 0.05 (∗), p < 0.01 (∗∗), p < 0.001 (∗∗∗). A significant strain × time interaction (F(6, 24) = 9.97, p < 0.001) indicates distinct temporal dynamics between strains.

The resulting strain *Synechocystis* Δ*inv* pSHDY_P*phoA*∗::*spsA* was pre-cultivated in medium containing 0.5 mM phosphate to suppress *spsA* expression in the preculture. For the sucrose production process, a continuous cultivation was chosen. Therefore, the cells were grown to an optical density of 1 in medium containing 0.1 mM phosphate and subsequently kept at this density for the production phase by automated dilution with medium containing 0.05 mM phosphate (Fig. 5A). Using this strategy, sucrose production could be increased 5.5-fold in the production phase compared to the growth phase, in which P*phoA*∗ was not yet activated (Fig. 5C). In contrast, constitutive *spsA* expression of a control strain in which *spsA* was controlled by the constitutively active J23101 promoter led to an unregulated sucrose production over the complete process which was 10-fold lower in the production phase compared to the P*phoA*∗-driven expression. Thereby, the growth rates of both strains were comparable in the initial growth phase as well as in the denser cultures in the later production phase (Fig. 5B). This indicates that the potential metabolic burden by a 10-fold higher sucrose production could be avoided in the growth phase in which higher growth rates can be achieved compared to phases of higher cell densities.

### 3.6 Conservation of the *SphS-SphR system among cyanobacteria allows utilization of corresponding promoters from* Synechocystis *in other strains*

**Fig. 6:**
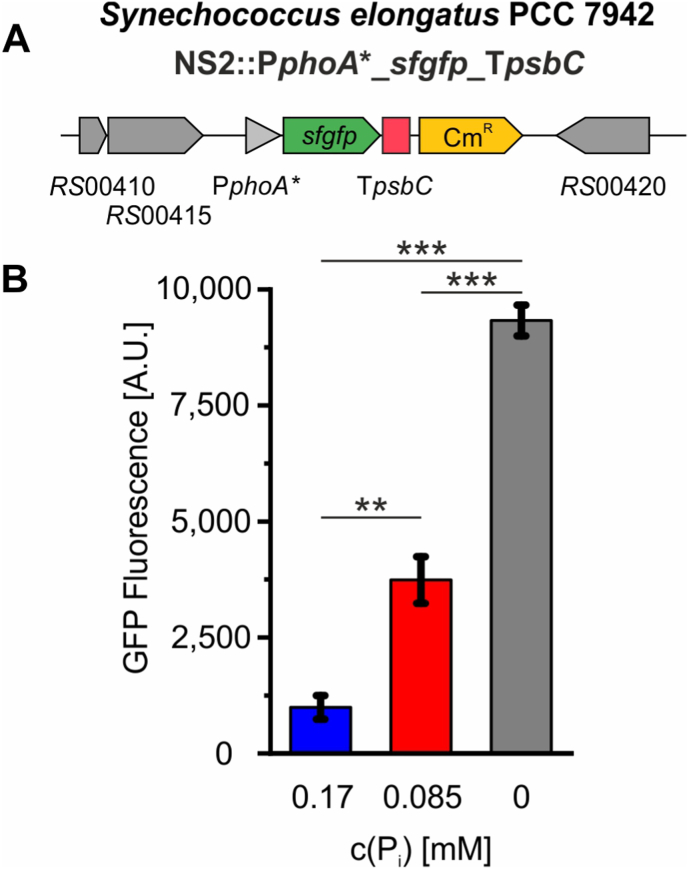
Phosphate-dependent activity of P*phoA*∗ from *Synechocystis* in *Synechococcus elongatus.* (A) The *phoA*∗ expression system from *Synechocystis* was transferred into neutral site 2 (NS2) of *Synechococcus elongatus* controlling a *sfgfp* as a reporter gene. (B) GFP fluorescence of the respective strain after two days in media with different phosphate concentrations. Cultures with media containing either 0.17 mM, 0.085 mM, or 0 mM phosphate were inoculated from a preculture with standard BG11 medium (0.17 mM phosphate) and incubated for two days before the GFP fluorescence was measured. Data are the mean and SE of three biological replicates with three technical replicates each. Fluorescence values of the WT under the same conditions were used for background correction. Differences between groups were analyzed using one-way ANOVA followed by Tukey's post hoc test. Significant differences are indicated as p > 0.05 (n.s.), p < 0.05 (∗), p < 0.01 (∗∗), p < 0.001 (∗∗∗).

The SphR response regulator and consequently its binding sites differ from the analogous system of *E. coli*. Hence, respective promoters could not be transferred from *E. coli* to *Synechocystis* or vice versa. Comparison of the SphR sequences across cyanobacteria revealed specific substitutions in positions responsible for DNA-motif recognition in PhoB of *E. coli* that are conserved among cyanobacteria (Fig. S4). Consequently, sequences resembling the SphR-binding motifs in *Synechocystis* can be found in the promoters for phosphate uptake related genes in other cyanobacterial strains (Su et al., 2007). This conservation of the SphS-SphR system among cyanobacteria potentially allows the application of the phosphate-responsive promoters from *Synechocystis* characterized and used here in other strains. To test the compatibility, the P*phoA*∗::*sfgfp* cassette was inserted into neutral site 2 (NS2) of *Synechococcus elongatus,* which is another frequently employed model organisms from a different cyanobacterial clade (Fig. 6A) (Clerico et al., 2007; Gupta and Mathews, 2010). The GFP fluorescence of the resulting strain was examined for its response to varying phosphate concentrations in the medium. Analogously to the response in *Synechocystis*, P*phoA*∗ triggered nine-fold increased *sfgfp* expression under phosphate limitation compared to phosphate-repleted conditions (Fig. 6B). Moreover, it already reacts to halfway reduced phosphate availability by 40 % of the maximal expression level achieved without phosphate. However, the overall expression strength was lower in *Synechococcus elongatus* compared to *Synechocystis*.

## 4 Discussion

Controlled gene expression is essential for biotechnological processes, and both constitutive and inducible promoter systems are widely used. Constitutive expression is simple but imposes a constant metabolic burden and increases selection pressure for escape mutations, whereas inducible systems enable temporal control but rely on additional components and costly chemical inducers, which become impractical at scale (Cardoso et al., 2020; Lee and Kim, 2015). Auto-inducible systems address these limitations by coupling expression to physiological cues, thereby reducing metabolic burden during growth while eliminating the need for external inducers. This is particularly relevant for phototrophic production processes in cyanobacteria, where low-value products from CO_2_ and sunlight require strictly minimized production costs (Appel et al., 2020; Santos-Merino et al., 2023; Sarsekeyeva et al., 2015; Toepel et al., 2023).

In this study, we developed a promoter library that enables auto-inducible gene expression in cyanobacteria based on the Pho regulon. This strategy allows gene expression to be directly coupled to the external phosphate concentration in the growth medium avoiding toxic or costly inducers. Moreover, the need for expressing additional regulatory proteins such as transcription factors is eliminated. Instead, the promoter library exploits the native SphS–SphR two-component system, which is conserved across cyanobacteria (Fig. S4), and responds to physiological changes that occur naturally during standard cultivation. Hence, transferability across cyanobacterial strains can be assumed and was demonstrated here for *Synechococcus elongatus* (Fig. 6). However, different expression strengths and induction characteristics compared to *Synechocystis* may arise. Similar Pho-regulon-based auto-inducible systems have been reported in *E. coli* and *Bacillus subtilis,* where promoter activation was associated with phosphate starvation and growth arrest (Gundinger et al., 2022; Kerovuo et al., 2000; Nguyen et al., 2019). In contrast, luxury uptake and phosphate storage in cyanobacteria enables activation of the SphS-SphR system prior to severe phosphate limitation (Griese et al., 2011; Xiao et al., 2022). Accordingly, promoter activation in *Synechocystis* remained compatible with continued growth, as demonstrated by sustained growth in P_i_-free medium (Fig. 3A) and during continuous cultivation in the sucrose production process (Fig. 6).

Maximal expression levels from the Pho-promoters depended not only on the current external P_i_ concentration but also on the initial P_i_ concentration supplied at the start of the cultivation (Fig. 2, Fig. 3, Fig. 4), likely reflecting the influence of intracellular P_i_ reserves accumulated during growth. Consequently, predictable auto-induction requires coordination of P_i_ profiles, biomass accumulation, and promoter choice in the process design. In batch cultures, the onset and strength of promoter activation can be controlled via the initial P_i_ concentration, with high P_i_ concentrations fully suppressing activation. In fed-batch processes, promoter activity can additionally be adjusted through the P_i_ concentration of the feed and the biomass concentration at feed initiation. In continuous or repeated-batch cultivations, the steady-state cell density further influences promoter activation through its effect on P_i_ consumption. Together, these control strategies enable induction without external inducers while maintaining tunability through process design. However, their dependence on environmental P_i_ concentrations may complicate applications in wastewater-based media or natural water sources where P_i_ concentrations can vary or may not be known (Usai et al., 2024).

When integrated into process design, the presented promoters enabled a robust auto-inducible transition from growth to production, as demonstrated in the proof-of-concept sucrose production process. Sucrose synthesis above basal activity (8.15 μmol∗L^−1^∗OD_750_^−1^) was initiated on the second cultivation day, shortly before the culture reached the target density for continuous operation. After four days, a 5.5-fold higher average sucrose concentration of 45 μmol∗L^−1^∗OD_750_^−1^ was obtained, corresponding to a production rate of ∼0.23 mg∗L^−1^∗h^−1^∗OD_750_^−1^. Previous studies on sucrose production in *Synechocystis* employed the copper inducible promoter of *petE* achieving concentrations of up to 25 mg∗L^−1^∗OD_730_^−1^ (73 μmol∗L^−1^∗OD_750_^−1^), although inducibility was not explicitly demonstrated. Further engineering, including expression of a sucrose exporter, additional pathway enzymes, and deletion of competing pathways, increased sucrose accumulation to 35 mg∗L^−1^∗OD_730_^−1^ (102 μmol∗L^−1^∗OD_730_^−1^) (Du et al., 2013). In a subsequent study, strain optimization, including deletion of the invertase gene, resulted in sucrose titers of up to 127 μmol∗L^−1^∗OD_750_^−1^ after 7 days of cultivation (Kirsch et al., 2018). Using the IPTG-inducible construct A1lacO-1_RBS(*cpcB*) for *spsA* and sucrose exporter expression, substantially higher production rates of up to 273 mg∗L^−1^∗day^−1^ in a batch culture (∼7.6 mg∗L^−1^∗h^−1^∗OD_750_^−1^ considering the average cell density of ∼1.5 OD_750_ in the first day) were achieved (Thiel et al., 2019). Recently, production rates from this genetic setup were further increased to a maximal rate of 38.64 mg∗L^−1^∗h^−1^ (∼26 mg∗L^−1^∗h^−1^∗OD_750_^−1^ considering the average cell density of ∼1.5 OD_750_ in the first day) by additionally deleting competing pathways and carbon flux redirection via CfrA/PirC-mediated regulation. For this strain a minimal basal sucrose secretion was detected with a strong increase (>10 fold) upon addition of the inducer IPTG (Domínguez-Lobo et al., 2025). In comparison to P*petE*, *spsA* overexpression from P*phoA*∗ resulted in comparable but slightly lower intracellular sucrose accumulation, while providing clear auto inducibility. Prolonged cultivation and higher cell densities may, however, further enhance promoter activity and product titer in this system.

Direct comparisons to other strains or the A1lacO-1 promoter exhibiting a higher induction fold are impeded by the strong impact of additional genetic modifications, especially sucrose secretion. Notably, highly optimized sucrose-producing strains frequently exhibited reduced growth after induction due to the metabolic burden associated with sucrose secretion (Domínguez-Lobo et al., 2025; Yun et al., 2024). These strong inhibitions of growth verify the need for inducible activation of such systems. Together with the already promising intracellular sucrose accumulation by P*phoA*∗-controlled *spsA* expression, they prove the potential of the presented expression system. Beyond sucrose production, the presented promoters may be particularly useful in photobiotechnological process where product formation directly competes with biomass accumulation, such as hydrogen production (Appel et al., 2020; Gutthann et al., 2007).

Among the promoters tested, the strongest GFP expression was achieved using P*phoA*∗. After two days in P_i_-free medium, its expression level reached a similar range as the previously reported inducible system combining P*J23119* and the guanidine-responsive riboswitch (Itzenhäuser et al., 2025), although it remained approximately fivefold lower than the expression observed with the rhamnose-inducible promoter (Kelly et al., 2018) (see Table S3 for a comparison with other inducible expression systems).

For the *phoA* promoter, replacing the native ribosomal binding site with RBS∗ increased GFP formation by more than twofold, substantially contributing to the strong expression observed. However, analogous replacements of the native RBS in the other promoters did not enhance but instead reduced the maximal expression levels (Fig. 2). Relative expression levels achieved with different RBS were previously known to be dependent on the promoter and the expressed gene in *Synechocystis* (Englund et al., 2016; Thiel et al., 2018). This highlights that protein formation is not a simple additive function of promoter activity and RBS strength but is shaped by a complex interplay of the entire genetic context (Englund et al., 2016). In particular, interactions between the promoter-derived 5′UTR, the RBS sequence, and the coding sequence can affect mRNA secondary structure, ribosome accessibility, translation initiation efficiency, and mRNA stability. Introduction of RBS∗ may therefore have altered local mRNA structure in a way that reduced the overall expression efficiency in certain promoter contexts while it enhanced it in others.

While P*phoA*∗ exhibited the strongest expression, all promoters from the library enabled tunable, P_i_-responsive auto-induction. In contrast, other systems developed for auto-inducibility in cyanobacteria like the heterologous quorum-sensing systems established in *Synechococcus elongatus* yet rely on externally inducible promoters for their activation and exhibit a limited induction between low and high cell densities (Kokarakis et al., 2025) or activate only after weeks of cultivation triggered by nitrogen starvation and other unidentified factors as the stationary phase specific promoter P*ndbA*600 (Madsen et al., 2021). By selecting late-responding promoters such as P*pstS2*, the induction time point can be delayed while maintaining identical phosphate management. Conversely, negatively regulated promoters can be used to achieve a reversed expression profile with higher expression levels early in the cultivation. Notably, the synthetic negative regulated promoter P*P*_*i*_*-neg* presented strict deactivation under phosphate limitation, making it attractive for such applications (Fig. 2). All tested promoters exhibited a measurable basal activity under phosphate-repleted conditions. While a low basal expression level is acceptable for many applications, it may pose challenges for expressing toxic or growth-inhibitory genes. In such cases, combining a positively regulated phosphate-responsive promoter driving the target gene with P*P*_*i*_*-neg* controlling expression of a corresponding antisense RNA could potentially suppress unintended expression.

## 5 Conclusion

In this study, we established a set of phosphate-responsive promoters that enables robust and tuneable auto-inducible gene expression in cyanobacteria by exploiting the native SphS–SphR two-component system. These promoters allow induction without external chemical inducers, without additional regulatory components, and minimal physiological perturbation, thereby reducing process costs and genetic burden. The promoters differ in their induction timing, dynamic range, and maximal expression strength, offering a versatile toolbox for controlling gene expression in diverse biotechnological applications.

Our results further demonstrate that promoter activation is strongly influenced by the initial and current external phosphate concentrations, underscoring the importance of integrating phosphate dynamics into process design. When appropriately managed, these promoters support a reliable transition from a growth phase into a production phase, as shown by the successful auto-induced sucrose production.

Beyond *Synechocystis*, the conservation of SphR suggests broad transferability of this system to other cyanobacterial species. Overall, this work expands the genetic toolbox for cyanobacterial biotechnology with auto-inducible promoters and provides a foundation for developing low-cost and scalable, photobiotechnological processes.

## CRediT authorship contribution statement

**Marvin Amadeus Itzenhäuser:** Writing – review & editing, Writing – original draft, Visualization, Supervision, Methodology, Investigation, Formal analysis, Conceptualization. **Franziska Hufnagel:** Writing – review & editing, Investigation, Data curation. **Carla Klemm:** Investigation, Data curation. **Kevin Otec:** Writing – review & editing, Investigation, Data curation. **Minmin Pan:** Methodology. **Stephan Klähn:** Writing – review & editing, Supervision, Funding acquisition, Formal analysis, Conceptualization.

## Declaration of generative AI and AI-assisted technologies in the manuscript preparation process

The manuscript was drafted by the authors. ChatGPT 5.1 was used only for language editing and stylistic refinement of pre-existing text. The authors take full responsibility for the content of the published article.

## Funding

The UFZ is supported by the European Regional Development Funds (EFRE, Europe funds Saxony) and the Helmholtz Association. This research was funded by the Helmholtz Association and the Deutsche Forschungsgemeinschaft (individual grant KL 3114/7-1 obtained by S.K.).

## Declaration of competing interest

The authors declare the following financial interests/personal relationships which may be considered as potential competing interests

Stephan Klaehn reports financial support was provided by German Research Foundation. If there are other authors, they declare that they have no known competing financial interests or personal relationships that could have appeared to influence the work reported in this paper.

## Data Availability

Data will be made available on request.
