# Supplementary material for: Utilization of phosphate-responsive promoters for auto-inducible management of biotechnological processes based on cyanobacteria
Source: Metab Eng Commun. 2026 May 22;22:e00278. doi: 10.1016/j.mec.2026.e00278 (PMC13226834; doi:10.1016/j.mec.2026.e00278)
Supplement: Multimedia component 1 [file mmc1.docx]

**Appendix**


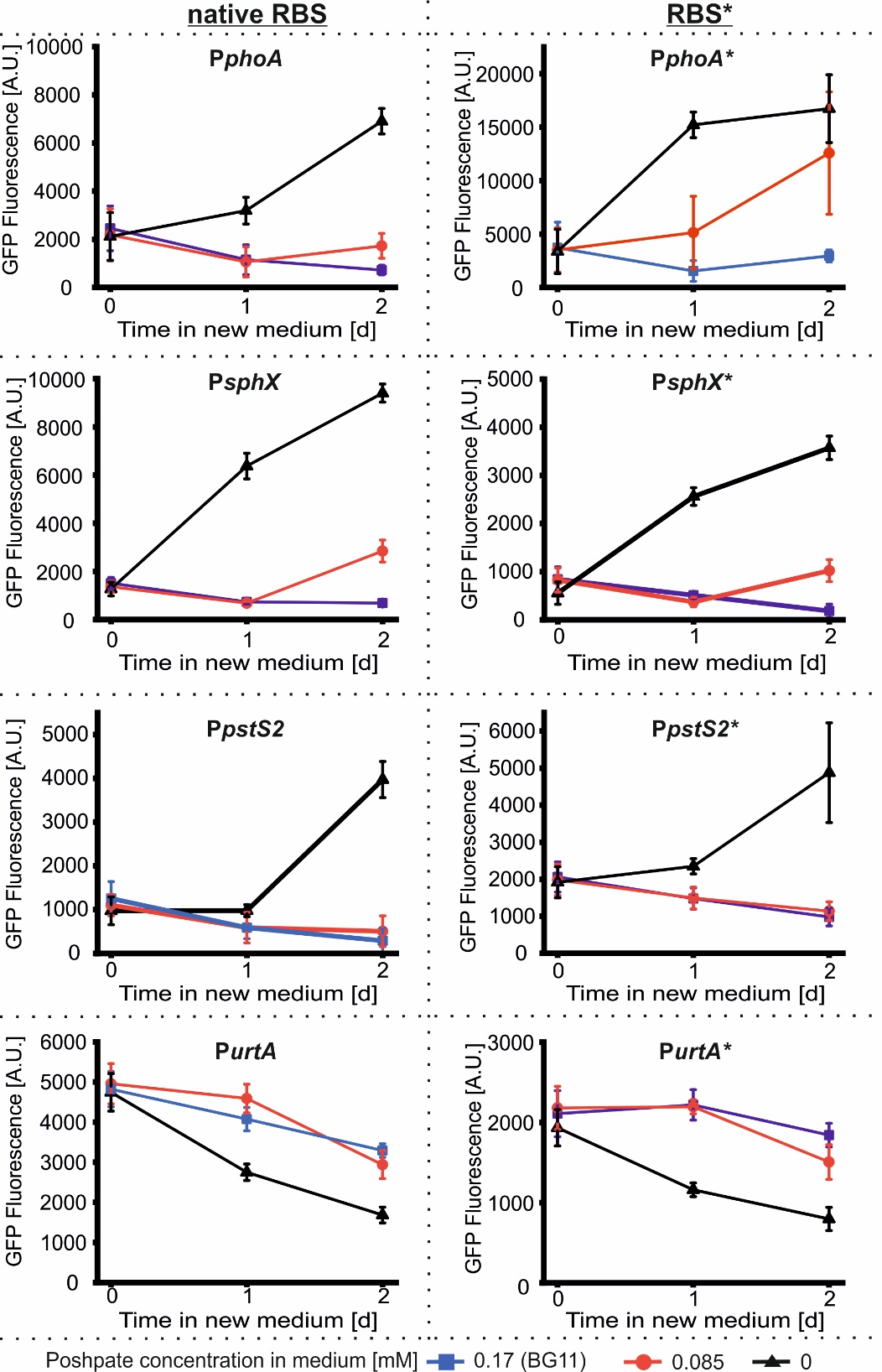


**Fig. S1: Temporal profile of native Pho-regulon promoters from *Synechocystis* with native or synthetic ribosomal binding site in different phosphate concentrations*.*** *Synechocystis* strains carrying plasmids harboring the respective promoter fused to *sfgfp* were analyzed for their GFP fluorescence level. Expression of GFP was observed for two days after a transfer from BG11 medium with 0.17 mM phosphate into to media with either 0.17 mM, 0.085 mM, or 0 mM phosphate. Data represents the averages and standard error of the means of three biological replicates from independent colonies. Fluorescence values of a strain harboring an empty vector were subtracted as background. Fold changes and representative absolute GFP levels presented in **Fig. 2** are based on this data.


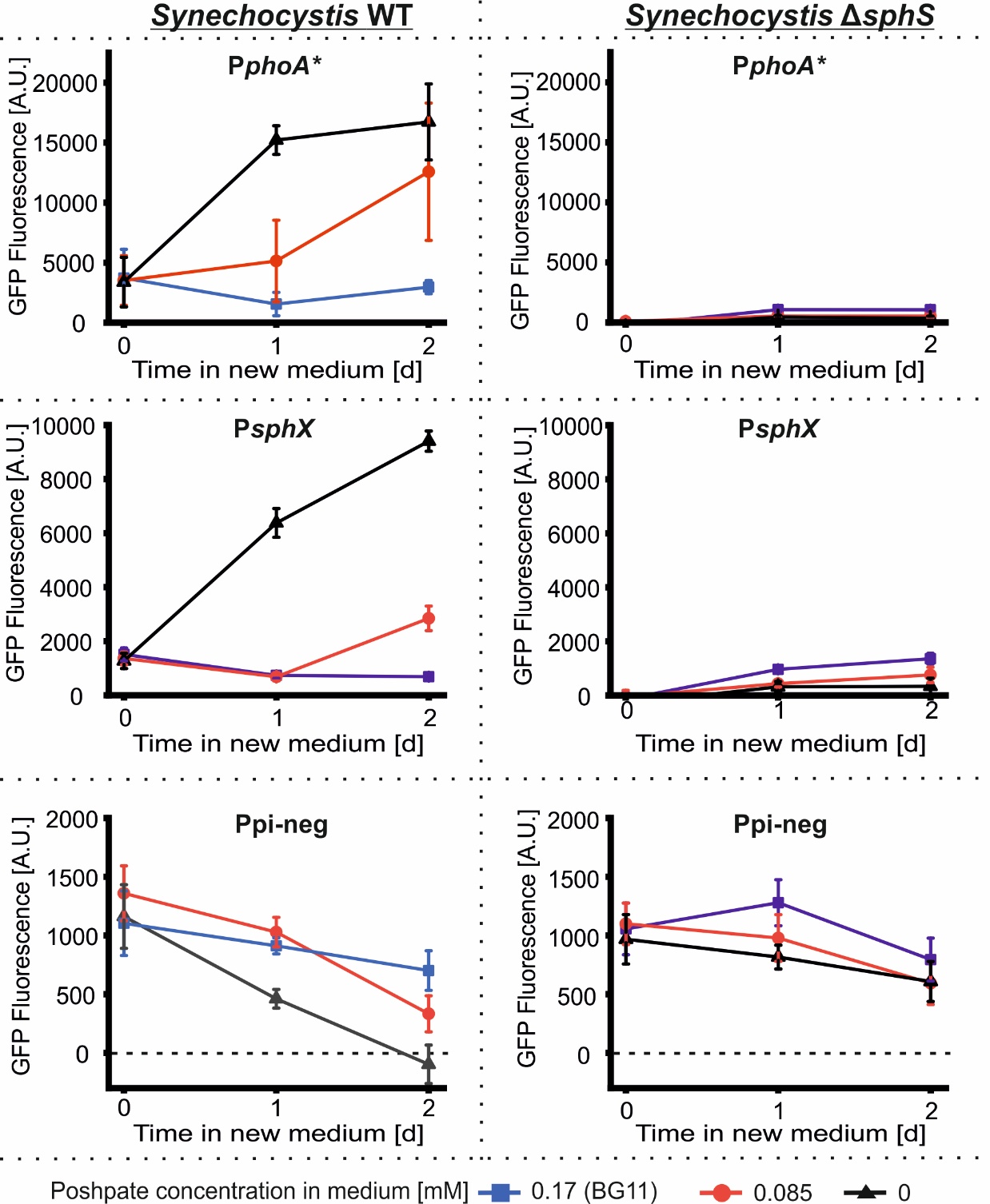


**Fig. S2: Temporal profile of Pho-regulon promoters in different phosphate concentrations in WT and *sphS* deletion mutant*.*** *Synechocystis* WT or *Synechocystis* Δ*sphS* strains carrying plasmids harboring the respective promoter fused to *sfgfp* were analyzed for their GFP fluorescence level. Expression of GFP was observed for two days after a transfer from BG11 medium with 0.17 mM phosphate into media with either 0.17 mM, 0.085 mM, or 0 mM phosphate. Data represents the averages and standard error of the means of three biological replicates from independent colonies. Fluorescence values of a strain harboring an empty vector were subtracted as background. Fold changes and representative absolute GFP levels presented in **Fig. 2** are partially based on this data.


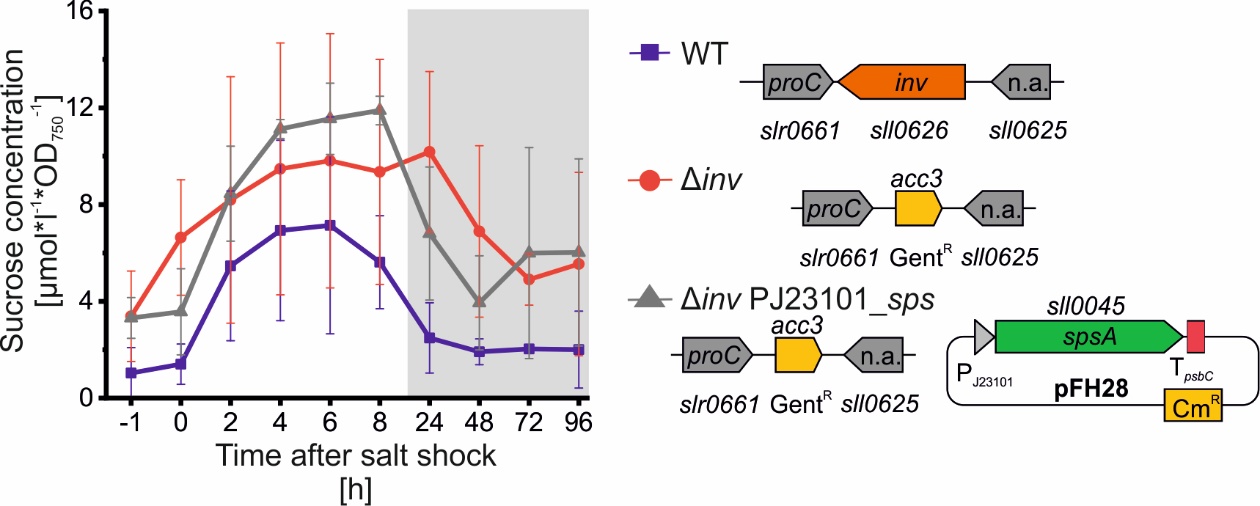


**Fig. S3: Sucrose production dynamic of different *Synechocystis* mutants after salt shock*.*** Respective *Synechocystis* strains were precultured in BG11 medium and transferred into BG11 medium supplemented with 500 mM NaCl at t = 0h. Sucrose levels were analyzed after extraction from the cells. Data represent the average and standard deviation of at least three replicates. Please note the difference in the scale of the x-axis after 8h (grey area). *Synechocystis* Δ*inv* PJ23101_*spsA* was employed as a control strain for constitutive SPS expression in the experiment presented in **Fig. 6**.


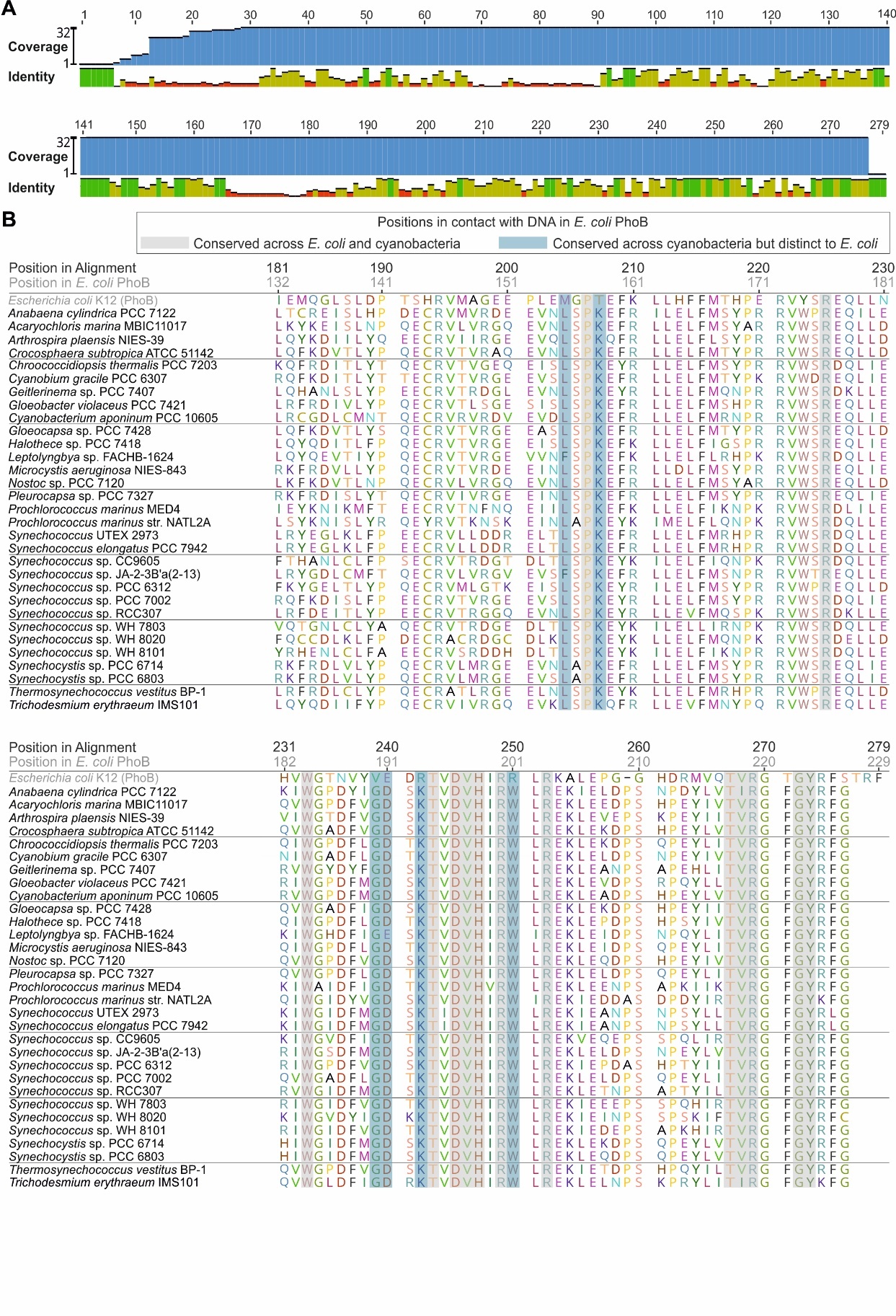


**Fig. S4: Sequence analysis of SphR proteins in cyanobacteria*.*** Orthologous of the SphR protein of *Synechocystis* in 30 representative cyanobacteria were extracted from the NCBI database using the reciprocal best hit method (1) and aligned together with PhoB of *E. coli*. (A) Coverage and sequence identity for the alignment across the entire protein. (B) Aligned sequences of the DNA binding region. Positions with direct contact to DNA in PhoB of *E. coli* according to Yamane et al. (2008) are highlighted (2).

**Table S1: Sequence of primers used in this study.** The primer binding regions are given in bold, BpiI recognition sites are marked yellow, BsaI recognition sites are marked green, LguI recognition sites are marked cyan, cutting sites of the restriction enzymes are underlined.

| number | Primer name | Sequence 5´ -> 3´ | Primer target |
| --- | --- | --- | --- |
| P1 | sfgfp for 1 | AAAGGTCTCGG**ATGAGCAAAGGAGAAGAACTTTTC** | *sfgfp* |
| P2 | sfgfp for 2 | AAAGGTCTCGATGA**GCAAAGGAGAAGAACTTTTC** | *sfgfp* |
| P3 | sfgfp rev | AAAGGTCTCGCTTT**TTATTTGTAGAGCTCATCCATGC** | *sfgfp* |
| P4 | PphoA for | AAAGGTCTCGGATG**TTTTCGTTCCCCGTTGACAATG** | *phoA* promoter |
| P5 | PphoA rev | AAAGGTCTCGTCAT**AATTGCTTTAGAAATTTCTCAATCAGG** | *phoA* promoter |
| P6 | PphoA* rev | AAAGGTCTCGCTTT**AATTGCTTTAGAAATTTCTCAATCAGG** | *phoA* promoter |
| P7 | PsphX for | AAAGGTCTCGGATG**GACTGGAATTACGGCTCGAAG** | *sphX* promoter |
| P8 | PsphX rev | AAAGGTCTCGTCAT**TTTTACTTCTCCGCCCCCAG** | *sphX* promoter |
| P9 | PsphX* rev | AAAGGTCTCGCTTT**GCCCCCAGGGTGGTCG** | *sphX* promoter |
| P10 | PpstS2 for | AAAGGTCTCGGATG**CAGTTTTTTTCAAGGGATAACCAC** | *pstS2* promoter |
| P11 | PpstS2 rev | AAAGGTCTCGTCAT**AGGAATTTGCAATCTAAATTGTAGTG** | *pstS2* promoter |
| P12 | PpstS2* rev | AAAGGTCTCGCTTT**AGGAATTTGCAATCTAAATTGTAGTG** | *pstS2* promoter |
| P13 | PurtA for | AAAGGTCTCAGATG**TTCCCCAATAACTAGCCC** | *urtA* promoter |
| P14 | PurtA rev | AAAGGTCTCATCAT**ACGCGAAAAATTATTACCTCG** | *urtA* promoter |
| P15 | PurtA* rev | AAAGGTCTCACTTT**ACGCGAAAAATTATTACCTCG** | *urtA* promoter |
| P16 | PJ23119 rev | AAAGGTCTCGTCATCTAGTATTTCTCCTCTTTGCTAGC**ATTATACCTAG** | P17, P18, P19 |
| P17 | PJ23119 for | AAAGGTCTCGGATGTTGACAGCTAGCTCAGTC**CTAGGTATAAT** | P16 |
| P18 | Ppi-neg for | AAAGGTCTCGGATGTTGACATAATCTTTTCTTAATCT**CTAGGTATAAT** | P16 |
| P19 | 3’ hom sphS for | AAAGGTCTCGGATG**AATTCGACCAACAGAAGGTCAAG** | downstream of *sphS* |
| P20 | 3’ hom sphS rev | AAAGGTCTCGCTTT**TGAGGAATTTTTCCAACGTTTAACC** | downstream of *sphS* |
| P21 | 5’ hom sphS for | AAAGGTCTCGGATG**TGTGTTGACCTACACCAAGG** | upstream of *sphS* |
| P22 | 5’ hom sphS rev | AAAGGTCTCGCTTT**CCCATTGAATTCTAGTCTTTTTAAG** | upstream of *sphS* |
| P23 | 5’ hom 7942 NS2 for | AAAGCTCTTCAGTCGGTCTCAGCCA**TAGATGAGGAAGCATGAGCG** | upstream of NS2 (*Syn. elongatus* PCC7942) |
| P24 | 5’ hom 7942 NS2 rev | AAAGCTCTTCAGGTCTCTTAAC**CACCCATCCATGCACTGG** | upstream of NS2 (*Syn. elongatus* PCC7942) |
| P25 | 3’ hom 7942 NS2 for | AAAGCTCTTCAGTCGGTCTCAGAGC**TGCAATGACGGACAGAGGC** | downstream of NS2 (*Syn. elongatus* PCC7942) |
| P26 | 3’ hom 7942 NS2 rev | AAAGCTCTTCAGGTCTCTTCCT**ATTTTCGACTGTGATGGTGTGC** | downstream of NS2 (*Syn. elongatus* PCC7942) |
| P27 | 7942 NS2 verification for | **CATCAGATCGATGCGATCG** | upstream of 5’hom. region of NS2 (*Syn. elongatus* PCC7942) |
| P28 | pUC LguI for | AAAGGTCTCTCATC**TTAAGCCAGCCCCGACAC** | pUC18 |
| P29 | pUC LguI rev | AAAGGTCTCTTGGC**TTCCTCGCTCACTGACTCG** | pUC18 |
| P30 | lacZ LguI for | AAAGGTCTCTGCCAGTCAGAAGAGC**GTGCGCAACGCAATTAATG** | *lac* promoter |
| P31 | lacZ LguI rev | AAAGGTCTCTGATGGGTAGAAGAGC**CCTCGAGCTATGCGGC** | *lacZ* |
| P32 | 5’ hom lim17 for | TTTGCTCTTCTGTCGGTCTCTGC**CAAACTGCTCCGCTGTTGC** | upstream of *lim17* |
| P33 | 5’ hom lim17 rev | TTTGCTCTTCTGGTCTCTTCTG**ATTCCGTTGTCCATTTATTTTTTGCC** | upstream of *lim17* |
| P34 | 3’ hom lim17 for | TTTGCTCTTCTGTCGGTCTCTTGTG**AATTTTCCTGAACTAGTCTGACG** | downstream of *lim17* |
| P35 | 3’ hom lim17 rev | TTTGCTCTTCTGGTCTCTGATG**AAAGCTTGTCTGACCTTGCC** | downstream of *lim17* |
| P36 | lim17 deletion verification for | **GAAACGGTGTTGGCCTTGG** | upstream of 5’hom. region of *lim17* |
| P37 | in lim17 rev | **CCATTCCCCAATCGGATCAC** | *lim17* |
| P38 | GentR for | AAAGGTCTCGGATG**CCGTTGCGCTGCCC** | Gent^R^ |
| P39 | GentR rev | AAAGGTCTCTTCTGCTTT**GCCGAGCACCTGGTCGC** | Gent^R^ |
| P40 | Tdouble for | AAAGGTCTCTCAGAGATG**CCAGGCATCAAATAAAACG** | Tdouble – Bba_B0015 |
| P41 | Tdouble rev | AAAGGTCTCTCTTT**TATAAACGCAGAAAGGCCCAC** | Tdouble – Bba_B0015 |
| P42 | PJ23101 for | AAAGGTCTCGGATGTTTACAGCTAGCTC**AGTCCTA** | P43 |
| P43 | PJ23101 rev | AAAGGTCTCGCTTTGCTAGCATAATACC**TAGGACT** | P42 |
| P44 | spsA for | AAAGGTCTCAG**ATGAGCTATTCATCAAAATAC** | *spsA* |
| P45 | spsA rev | AAAGGTCTCGCTTT**TTAAACGGGGTCTAACAA** | *spsA* |

**Table S2: List of plasmids used and generated in this study.**

Plasmids were generated using Golden Gate Assembly (GGA) in which the given elements were fused via their BsaI, BpiI, or LguI cutting sites. Elements were added to the reactions as plasmids or PCR products. PCR products are referred to as ‘PCR’ with the mentioning of the used primer pair as Pxx/Pxx and the used DNA template in brackets. If no template is mentioned, a PCR without a template yielding primer dimers resembling the desired sequence was carried out.

| **Vector** | **Level** | **5' overhang** | **3' overhang** | **Backbone** | **Selection** | **Plasmids or amplicons used in GGA to create the plasmid** | **Characteristics** | **Reference / source** |
| --- | --- | --- | --- | --- | --- | --- | --- | --- |
| **pGGC0** | 0 | GATG | AAAG | pUC18 | Amp |  | Level 0 empty entry vector based on pUC18 vector with additional integrated *BpiI/BsaI* restriction sites flanking *lacZα* | (3) |
| **pGGC1** | 1 | GCCA | GTTA | pUK21 | Kan |  | Level 1 position 1; empty entry vectors based on pUK21 vector with additional integrated BsaI/BpiI restriction sites flanking *lacZα* | (3) |
| **pGGC2.0** | 1 | GTTA | AAAG | pUK21 | Kan |  | Level 1 position 2.0; empty entry vectors based on pUK21 vector with additional integrated BsaI/BpiI restriction sites flanking *lacZα* | (3) |
| **pGGC2.3** | 1 | GTTA | CTAG | pUK21 | Kan |  | Level 1 position 2.3; empty entry vectors based on pUK21 vector with additional integrated BsaI/BpiI restriction sites flanking *lacZα* and RBS* | (3) |
| **pGGC3.0** | 1 | AAAG | CAGA | pUK21 | Kan |  | Level 1 position 3.0; empty entry vectors based on pUK21 vector with additional integrated BsaI/BpiI restriction sites flanking *lacZα* | (4) |
| **pGGC3** | 1 | CTAG | CAGA | pUK21 | Kan |  | Level 1 position 3; empty entry vectors based on pUK21 vector with additional integrated BsaI/BpiI restriction sites flanking *lacZα* | (3) |
| **pGGC4** | 1 | CAGA | TGTG | pUK21 | Kan |  | Level 1 position 4; empty entry vectors based on pUK21 vector with additional integrated BsaI/BpiI restriction sites flanking *lacZα* | (3) |
| **pGGC5** | 1 | TGTG | GAGC | pUK21 | Kan |  | Level 1 position 5; empty entry vectors based on pUK21 vector with additional integrated BsaI/BpiI restriction sites flanking *lacZα* | (3) |
| **pGGC21** | 1 | CAGA | TGTG | pGGC4 | Kan |  | Level 1 position 4 T*_psbC_* | (3) |
| **pGGC40** | 1 | GCCA | GTTA | pUK21 | Kan |  | End-linker level 1 spanning position 1 till 2 based on pUK21 vector with additional integrated *BsaI/BpiI* restriction sites flanking end-linker sequence TCGGTCACATGTGCATCCTCGATCTCA | (3) |
| **pGGC42** | 1 | CAGA | CATC | pUK21 | Kan |  | End-linker level 1 spanning position 4 till 7 based on pUK21 vector with additional integrated *BsaI/BpiI* restriction sites flanking end-linker sequence TCGGTCACATGTGCATCCTCGATCTCA | (4) |
| **pGGC43** | 1 | GAGC | CATC | pUK21 | Kan |  | End-linker level 1 spanning position 5 till 7 based on pUK21 vector with additional integrated *BsaI/BpiI* restriction sites flanking end-linker sequence TCGGTCACATGTGCATCCTCGATCTCA | (3) |
| **pGGC46** | 2 | GCCA | CATC | pBluescript II SK (+) | Amp |  | Level 2 empty entry vector based on pBluescript II SK (+) vector with additional integrated *BsaI* restriction sites flanking *lacZα* | (3) |
| **pGGC48** | 2 | GCCA | CATC | pBluescript II SK (+) | Cmc |  | Level 2 empty entry vector based on pBluescript II SK (+) vector with additional integrated *BsaI* restriction sites flanking *lacZα* | (4) |
| **pGGC64** | 0 | GATG | AAAG | pGGC0 | Amp |  | Level 0 Cmc^R^ | (4) |
| **pGGC105** | 1 | TGTG | GAGC | pGGC5 | Kan, Cmc | pGGC5; pGGC64 | Level 1 position 5 Cmc^R^ | This study |
| **pGGC206** | 2 | GCCA | CATC | pSHDY | Cmc |  | Level 2 empty entry vector based on pSHDY backbone; Spec^R^ exchanged with Cm^R^; integrated *lacZα* flanked by *BsaI* restriction sites | This study |
| **pGGC286** | 1 | GTTA | CTAG | pUK21 | Kan | pGGC2.3; pGGC0_PJ23101 | Level 1 position 2.3 PJ23101_RBS* | This study |
| **pGGC335** | 0 | GATG | AAAG | pGGC0 | Amp |  | Level 0 Kan^R^::T*tonB* | (4) |
| **pGGC336** | 1 | AAAG | CAGA | pGGC3.0 | Kan |  | Level 1 Position 3.0 T*psbC* | (4) |
| **pGGC341** | 0 | GATG | AAAG | pGGC0 | Amp | pGGC0; PCR P38/P39 (pSOMA17); PCR P40/P41 (pSEVA351_sfGFP) | Level 0 Gent^R^::Tdouble | This study |
| **pGGC342** | 1 | CAGA | TGTG | pUK21 | Kan | pGGC4; pGGC341 | Level 1 position 4 Gent^R^::Tdouble | This study |
|  |  |  |  |  |  |  |  |  |
| **pAI14** | 0 | GATG | AAAG | pGGC0 | Amp | pGGC0; PCR P19/P20 (*Syn. 6803* gDNA)) | Level 0 - 3’ homologous region of sphS | This study |
| **pAI15** | 0 | GATG | AAAG | pGGC0 | Amp | pGGC0; PCR P21/P22 (*Syn. 6803* gDNA)) | Level 0 - 5’ homologous region of sphS | This study |
| **pAI20** | 0 | GATG | AAAG | pGGC0 | Amp | pGGC0; PCR P7/P9 (*Syn. 6803* gDNA)) | Level 0 P*_sphX_** | This study |
| **pAI21** | 0 | GATG | AAAG | pGGC0 | Amp | pGGC0; PCR P4/P6 (*Syn. 6803* gDNA)) | Level 0 P*_phoA_** | This study |
| **pAI22** | 0 | GATG | AAAG | pGGC0 | Amp | pGGC0; PCR P10/P12 (*Syn. 6803* gDNA)) | Level 0 P*_psS2_** | This study |
| **pAI23** | 0 | GATG | AAAG | pGGC0 | Amp | pGGC0; PCR P1/P3 (pXG10_SF) | Level 0 *sfgfp* | This study |
| **pAI24** | 0 | GATG | AAAG | pGGC0 | Amp | pGGC0; PCR P2/P3 (pXG10_SF); PCR P7/P8 (*Syn. 6803* gDNA) | Level 0 P*_sphX_*::*sfgfp* | This study |
| **pAI25** | 0 | GATG | AAAG | pGGC0 | Amp | pGGC0; PCR P2/P3 (pXG10_SF); PCR P4/P5 (*Syn. 6803* gDNA) | Level 0 P*_phoA_*::*sfgfp* | This study |
| **pAI26** | 0 | GATG | AAAG | pGGC0 | Amp | pGGC0; PCR P2/P3 (pXG10_SF); PCR P10/P11 (*Syn. 6803* gDNA) | Level 0 P*_pstS2_*::*sfgfp* | This study |
| **pAI27** | 1 | AAAG | CAGA | pGCC3.0 | Kan | pGGC3.0; pAI14 | Level 1 position 3.0 - 3’ homologous region of sphS | This study |
| **pAI29** | 1 | GCCA | GTTA | pGGC1 | Kan | pGGC1; pAI15 | Level 1 position 1 - 5’ homologous region of sphS | This study |
| **pAI59 -**  **pP*sphX*** | 2 | - | - | pSHDY | Cmc | pGGC206; pGGC40; pAI77; pGGC336; pGGC42 | *sphX* promoter with native RBS fused to *sfgfp* on pSHDY backbone | This study |
| **pAI60 -**  **pP*sphX**** | 2 | - | - | pSHDY | Cmc | pGGC206; pGGC40; pAI74; pAI84; pGGC21; pGGC43 | *sphX* promoter with RBS* fused to *sfgfp* on pSHDY backbone | This study |
| **pAI61 -**  **pP*phoA*** | 2 | - | - | pSHDY | Cmc | pGGC206; pGGC40; pAI78; pGGC336; pGGC42 | *phoA* promoter with native RBS fused to *sfgfp* on pSHDY backbone | This study |
| **pAI62 -**  **pP*phoA**** | 2 | - | - | pSHDY | Cmc | pGGC206; pGGC40; pAI75; pAI84; pGGC21; pGGC43 | *phoA* promoter with RBS* fused to *sfgfp* on pSHDY backbone | This study |
| **pAI63 -**  **pP*pstS2*** | 2 | - | - | pSHDY | Cmc | pGGC206; pGGC40; pAI79; pGGC336; pGGC42 | *pstS2* promoter with native RBS fused to *sfgfp* on pSHDY backbone | This study |
| **pAI64 -**  **pP*pstS2**** | 2 | - | - | pSHDY | Cmc | pGGC206; pGGC40; pAI76; pAI84; pGGC21; pGGC43 | *pstS2* promoter with RBS* fused to *sfgfp* on pSHDY backbone | This study |
| **pAI66** | 1 | GTTA | AAAG | pGGC2.0 | Kan | pGGC2.0; pGGC335 | Level 1 position 2.0 Kan^R^::T*tonB* | This study |
| **pAI74** | 1 | GTTA | CTAG | pGGC2.3 | Kan | pGGC2.3; pAI20 | Level 1 position 2.3 P*_sphX_** | This study |
| **pAI75** | 1 | GTTA | CTAG | pGGC2.3 | Kan | pGGC2.3; pAI21 | Level 1 position 2.3 P*_phoA_** | This study |
| **pAI76** | 1 | GTTA | CTAG | pGGC2.3 | Kan | pGGC2.3; pAI22 | Level 1 position 2.3 P*_pstS2_** | This study |
| **pAI77** | 1 | GTTA | AAAG | pGGC2.0 | Kan | pGGC2.0; pAI24 | Level 1 position 2.0 P*_sphX_*::*sfgfp* | This study |
| **pAI78** | 1 | GTTA | AAAG | pGGC2.0 | Kan | pGGC2.0; pAI25 | Level 1 position 2.0 P*_phoA_*::*sfgfp* | This study |
| **pAI79** | 1 | GTTA | AAAG | pGGC2.0 | Kan | pGGC2.0; pAI26 | Level 1 position 2.0 P*_pstS2_*::*sfgfp* | This study |
| **pAI84** | 1 | CTAG | CAGA | pGGC3 | Kan | pGGC3; pAI23 | Level 1 position 3 *sfgfp* | This study |
| **pAI126** | 0 | GATG | AAAG | pGGC0 | Amp | pGGC0; PCR P2/P3 (pXG10_SF); PCR P13/P14 (*Syn.* 6803 gDNA) | Level 0 P*_urtA_*::*sfgfp* | This study |
| **pAI128** | 1 | GTTA | AAAG | pGGC2.0 | Kan | pGGC2.0; pAI126 | Level 1 position 2.0 P*_urtA_*::*sfgfp* | This study |
| **pAI129** | 0 | GATG | AAAG | pGGC0 | Amp | pGGC0; PCR P13/P15 (*Syn. 6803* gDNA)) | Level 0 P*_urtA_** | This study |
| **pAI131 -**  **pP*urtA*** | 2 | - | - | pSHDY | Cmc | pGGC206; pGGC40; pAI126; pGGC336; pGGC42 | *urtA* promoter with native RBS fused to *sfgfp* on pSHDY backbone | This study |
| **pAI133** | 1 | GTTA | CTAG | pGGC2.3 | Kan | pGGC2.3; pAI129 | Level 1 position 2.3 P*_urtA_** | This study |
| **pAI134 -**  **pP*urtA**** | 2 | - | - | pSHDY | Cmc | pGGC206; pGGC40; pAI133; pAI84; pGGC21; pGGC43 | *urtA* promoter with RBS* fused to *sfgfp* on pSHDY backbone | This study |
| **pAI171** | - | GTC | ACC | pUC18 | Amp | PCR P28/P29 (pGGC0); PCR P31/32 (pGGC0) | pUC18 backbone with LguI sites flanking a *lacZα* | This study |
| **pAI254** | 0 | GATG | AAAG | pGGC0 | Amp | pGGC0; PCR P2/P3 (pXG10_SF); PCR P16/P17 (-) | Level 0 P_J23119_::*sfgfp* | This study |
| **pAI255** | 0 | GATG | AAAG | pGGC0 | Amp | pGGC0; PCR P2/P3 (pXG10_SF); PCR P16/P18 (-) | Level 0 P_Pi-neg_::*sfgfp* | This study |
| **pAI257** | 1 | GTTA | AAAG | pGGC2.0 | Kan | pGGC2.0; pAI254 | Level 1 position 2.0 P_J23119_::*sfgfp* | This study |
| **pAI258** | 1 | GTTA | AAAG | pGGC2.0 | Kan | pGGC2.0; pAI255 | Level 1 position 2.0 P*_P_*_i-neg_::*sfgfp* | This study |
| **pAI260** | 2 | - | - | pBluescript II SK (+) | Cmc, Kan | pGGC48; pAI29; pAI66; pAI27; pGGC42 | Recombination template for *sphS* deletion with Kan^R^::T*tonB* resistance cassette | This study |
| **pAI261 -**  **pPPi-neg** | 2 | - | - | pSHDY | Cmc | pGGC206; pGGC40; pAI258; pGGC336; pGGC42 | Pi-neg promoter with native RBS fused to *sfgfp* on pSHDY backbone | This study |
| **pAI263 -**  **pPJ23119** | 2 | - | - | pSHDY | Cmc | pGGC206; pGGC40; pAI257; pGGC336; pGGC42 | PJ23119 promoter with native RBS fused to *sfgfp* on pSHDY backbone | This study |
| **pAI289** | - | GCCA | GTTA | pAI171 | Amp | pAI171; PCR P23/P24 (*Syn*. 7942 gDNA) | 5’ homologous region of NS2 of *Synechococcus elongatus* 7942 | This study |
| **pAI290** | - | GAGC | AGGA | pAI171 | Amp | pAI171; PCR P25/P26 (*Syn*. 7942 gDNA) | 3’ homologous region of NS2 of *Synechococcus elongatus* 7942 | This study |
| **pAI292** | 2 | - | - | pGGC46 | Amp, Cmc | pGGC46; pAI289; pAI75; pAI84; pGGC21; pGGC105; pAI290 | P*phoA**::*sfgfp*::T*psbC*::Cm^R^ with homologous regions for integration into NS2 of *Synechococcus elongatus* PCC7942 | This study |
| **pSHDY_*P_rhaBAD_::mVenus _PJ23119-rhaS*** | - | - | - | pSHDY | Spec |  | *P_J23119_::rhaS, P_rhaBAD_* | (5) |
| **pXG10_SF** | - | - | - | pXG | Cmc |  | pSC101-based plasmid containing the *sfgfp* reporter gene | (6) |
| **pSEVA351-*sfgfp*** | - | - | - | pSEVA 351 | Cmc^R^ |  | P_J23101__*sfgfp*_Tdouble | (7) |
| **pSOMA17** | - | - | - | pSOMA17 | Tdoub^R^ |  | Replicative plasmid based on the *Synechocystis* plasmid pCB2.4 | (7) |
| **pUC 5’-lim17** | - | GCCA | CAGA | pAI171 | Amp | pAI171; PCR P32/P33 (*Syn*. 6803 gDNA) | 5’ homologous region of *lim17* | This study |
| **pUC 3’-lim17** | - | TGTG | CATC | pAI171 | Amp | pAI171; PCR P34/P35 (*Syn*. 6803 gDNA) | 5’ homologous region of *lim17* | This study |
| **pGGC0_PJ23101** | 0 | GATG | AAAG | pGGC0 | Amp | pGGC0; PCR P42/P43 (-) | Level 0 J23101 promoter | This study |
| **pFH20** | 0 | GATG | AAAG | pGGC0 | Amp | pGGC0; PCR P44/P45 (*Syn*. 6803 gDNA) | Level 0 *sps* | This study |
| **pFH24** | 1 | CTAG | CAGA | pUK21 | Kan | pGGC3; pFH20 | Level 1 position 3 *sps* | This study |
| **pFH28** | 2 | - | - | pGGC206 | Cmc | pGGC206; pGGC40; pGGC286; pFH24; pGGC21; pGGC43 | Sucrose phosphate synthase (*spsA*) gene controlled by P_J23101_ on pSHDY backbone | This study |
| **pFH30** | 2 | - | - | pGGC206 | Cmc | pGGC206; pGGC40; pAI75; pFH24; pGGC21; pGGC43 | Sucrose phosphate synthase (*spsA*) gene controlled by P*_phoA_*_*_ on pSHDY backbone | This study |
| **pFH32** | 2 | - | - | pGGC48 | Cmc, Gent | pGGC48; pUC 5’-lim17; pGGC342; pUC 3’-lim17 | Recombination template for *lim17* deletion with Gent^R^::Tdouble resistance cassette | This study |

**Table S3: Comparison of phosphate-controlled gene expression with previously published inducible expression systems in Synechocystis.**

Data from literature has been extracted or estimated from figures if not available as numbers. Respective reporters used in the published studies are also given. Data for expression systems marked in grey were collected under comparable conditions. sfGFP fluorescence values from this study were extracted from **Fig. 2.** Standard errors are given as ±-values.

| **Expression system** | **Inducer** | **Reporter** | **Signal**  Non-induced conditions | **Signal**  Induced conditions | **Induction fold** | **Source** |
| --- | --- | --- | --- | --- | --- | --- |
| P*_phoA_* | P_i_ level | sfGFP | 1,154 ±620 | 6,905 ±529 | 6 | This study |
| P*_phoA_** | P_i_ level | sfGFP | 1,550 ±560 | 16,722 ±1,830 | 11 | This study |
| P*_sphX_* | P_i_ level | sfGFP | 733 ±112 | 9,404 ±375 | 13 | This study |
| P*_sphX_** | P_i_ level | sfGFP | 510 ±68 | 3,571 ±244 | 7 | This study |
| P*_pstS2_* | P_i_ level | sfGFP | 589 ±253 | 3,972 ±413 | 7 | This study |
| P*_pstS2_** | P_i_ level | sfGFP | 1,479 ±286 | 4,878 ±1,347 | 3 | This study |
| P*_urtA_* | P_i_ level | sfGFP | 4,074 ±290 | 1,685 ±197 | 0,4 | This study |
| P*_urtA_** | P_i_ level | sfGFP | 2,217 ±189 | 796 ±145 | 0,4 | This study |
| P_Pi-neg_ | P_i_ level | sfGFP | 911 ±69 | -96 ±164 | On/Off | This study |
| P_J23109__R_Gd_ | Guanidine | sfGFP | 51 ±131 | 562 ±234 | 11 | (4) |
| P_J23100__R_Gd_ | Guanidine | sfGFP | 114 ±216 | 762 ±335 | 7 | (4) |
| P_J23101__R_Gd_ | Guanidine | sfGFP | 217 ±144 | 3,341 ±279 | 15 | (4) |
| P_cpc__R_Gd_ | Guanidine | sfGFP | 1,087 ±882 | 9,237 ±883 | 9 | (4) |
| P_J23119__R_Gd_ | Guanidine | sfGFP | 847 ±175 | 21,462 ±1,245 | 25 | (4) |
| P_rha__R_Gd_ | Guanidine + Rhamnose | sfGFP | -199 ±187 | 3,110 ±669 | Off/On | (4) |
| P_rha_ | Rhamnose | sfGFP | 498 ±141 | 86,288 ±2,940 | 162 | (4) |
| P_rha_ | Rhamnose | YFP | ~175 | ~3000 | ~17 | (8) |
| P_trc__R_Theo_ [E*] | Theophylline | GFP | ~2000 | ~43,000 | 30 | (9) |
| P_nrsB_ | Nickel ions | EYFP | ~1.2 | ~53 | 39 | (10) |
| P_L03_ | Anhydrotetracycline | EYFP | 0.220 ±0.003 | 19.2 ±0.1 | 83 | (11) |
| P_trc10-core_ | IPTG | EYFP | ~16,380 | ~35,350 | 2.9 | (12) |
| P_vanCC_ | Vanillate | mVenus | ~400 | ~8,500 | 16 | (5) |
| LuxR/LuxI | Quorum sensing (0.05 mM IPTG) | mNG | ~7,500 | ~30,000 | 4 | (13) |
| LuxR/LasI | Quorum sensing (0.03 mM IPTG) | mNG | ~27,500 | ~55,000 | 2 | (13) |
| P*_ndbA_*_600_ | Auto-inducible / nitrogen | GFP | ~150 | ~1,600 | 11 | (14) |

# Supplementary References

1. A. R. Mushegian, E. V. Koonin, A minimal gene set for cellular life derived by comparison of complete bacterial genomes. *Proc. Natl. Acad. Sci. U. S. A.* **93**, 10268–10273 (1996).

2. T. Yamane, H. Okamura, M. Ikeguchi, Y. Nishimura, A. Kidera, Water-mediated interactions between DNA and PhoB DNA-binding/transactivation domain: NMR-restrained molecular dynamics in explicit water environment. *Proteins Struct. Funct. Bioinforma.* **71**, 1970–1983 (2008).

3. S. Lupacchini, *et al.*, Co-expression of auxiliary genes enhances the activity of a heterologous O_2_-tolerant hydrogenase in the cyanobacterium *Synechocystis* sp. PCC 6803. *Biotechnol. Biofuels Bioprod.* **18**, 41 (2025).

4. M. A. Itzenhäuser, *et al.*, Deciphering guanidine assimilation and riboswitch-based gene regulation in cyanobacteria for synthetic biology applications. *Proc. Natl. Acad. Sci.* **122**, e2519335122 (2025).

5. A. Behle, P. Saake, A. T. Germann, D. Dienst, I. M. Axmann, Comparative dose-response analysis of inducible promoters in cyanobacteria. *ACS Synth. Biol.* **9**, 843–855 (2020).

6. C. P. Corcoran, *et al.*, Superfolder GFP reporters validate diverse new mRNA targets of the classic porin regulator, MicF RNA. *Mol. Microbiol.* **84**, 428–445 (2012).

7. F. Opel, *et al.*, Generation of synthetic shuttle vectors enabling modular genetic engineering of cyanobacteria. *ACS Synth. Biol.* (2022). https://doi.org/10.1021/acssynbio.1c00605.

8. C. L. Kelly, G. M. Taylor, A. Hitchcock, A. Torres-Méndez, J. T. Heap, A Rhamnose-Inducible System for Precise and Temporal Control of Gene Expression in Cyanobacteria. *ACS Synth. Biol.* **7**, 1056–1066 (2018).

9. I. Cengic, I. C. Cañadas, N. P. Minton, E. P. Hudson, Inducible CRISPR/Cas9 Allows for Multiplexed and Rapidly Segregated Single-Target Genome Editing in *Synechocystis* Sp. PCC 6803. *ACS Synth. Biol.* **11**, 3100–3113 (2022).

10. E. Englund, F. Liang, P. Lindberg, Evaluation of promoters and ribosome binding sites for biotechnological applications in the unicellular cyanobacterium *Synechocystis* sp. PCC 6803. *Sci. Rep.* **6**, 36640 (2016).

11. H.-H. Huang, P. Lindblad, Wide-dynamic-range promoters engineered for cyanobacteria. *J. Biol. Eng.* **7**, 10 (2013).

12. D. Camsund, T. Heidorn, P. Lindblad, Design and analysis of LacI-repressed promoters and DNA-looping in a cyanobacterium. *J. Biol. Eng.* **8**, 4 (2014).

13. E. J. Kokarakis, M. Santos-Merino, S. Ghaffarinasab, D. Vocelle, D. C. Ducat, Engineering quorum-sensing circuits in *Synechococcus elongatus* PCC 7942 towards self-inducible systems. *Metab. Eng.* **92**, 76–89 (2025).

14. M. A. Madsen, G. Hamilton, P. Herzyk, A. Amtmann, Environmental Regulation of PndbA600, an Auto-Inducible Promoter for Two-Stage Industrial Biotechnology in Cyanobacteria. *Front. Bioeng. Biotechnol.* **8** (2021).
